# New Indomalayan *Nebularmis* species (Heterotardigrada: Echiniscidae) provoke a discussion on its intrageneric diversity

**DOI:** 10.1186/s40851-021-00172-0

**Published:** 2021-04-12

**Authors:** Piotr Gąsiorek, Katarzyna Vončina, Joanna Ciosek, Mariana Veloso, Paulo Fontoura, Łukasz Michalczyk

**Affiliations:** 1 Department of Invertebrate Evolution, Institute of Zoology and Biomedical Research, Faculty of Biology, Jagiellonian University, Gronostajowa 9, 30-387 Kraków, Poland; 2 Department of Biology, Faculty of Sciences, University of Porto, Rua Campo Alegre s/n, FC4, 4169-007 Porto, Portugal; 3 MARE, Marine and Environmental Sciences Centre, ISPA – Instituto Universitário, Rua Jardim do Tabaco, 34, 1149-041 Lisbon, Portugal

**Keywords:** Cuticle, Morphology, Oriental region, Phylogeny, Pseudocryptic species, Taxonomy

## Abstract

Recent years have brought undeniable progress in tardigrade taxonomy, and speciose complexes were detected in a number of phylogenetic lineages. The family Echiniscidae is one such lineage; it is one of the most diverse groups of limno-terrestrial tardigrades and can be characterized as having achieved great evolutionary success. In this contribution, using populations representing several species that originated from the Indomalayan region, we reconstructed phylogenetic affinities within *Nebularmis*, a recently erected genus within the *Echiniscus* lineage. *Nebularmis auratus* **sp. nov.** and *Nebularmis burmensis* **sp. nov.** are described from the Eastern Yoma Mountains and the Shan Hills (Myanmar), *Nebularmis bhutanensis* **sp. nov.** is described from the Eastern Himalayas (Bhutan), and *Nebularmis indicus* **sp. nov.** is described from the foothills of the Western Ghats (Goa, India). Moreover, males are reported in populations of the last two species. All known members of the genus can be phenotypically differentiated based on minute details of their dorsal sculpture and claws. Moreover, a very wide tropical distribution is demonstrated for *Nebularmis cirinoi*, recorded for the first time from islands of the Malay Archipelago. Furthemore, novel morphological, genetic, and geographic data allowed for the clarification of the generic diagnosis. Currently available data favor a scenario under which *Nebularmis* evolved in Southeast Asia and later dispersed to other regions of the globe.

**Supplementary Information:**

The online version contains supplementary material available at 10.1186/s40851-021-00172-0.

## Background

The diversity of tardigrades has been intensively studied in recent years using modern species delimitation tools, including statistical morphometry and molecular analyses. Consequently, numerous new species have been detected in every major lineage of the phylum, and many are currently awaiting formal descriptions. Among marine heterotardigrades, examples include *Echiniscoides* and *Batillipes* [1, 2], terrestrial heterotardigrade examples (predominantly echiniscids) are present in *Pseudechiniscus* and *Hypechiniscus* [3, 4], in the eutardigrade order Apochela, the genus *Milnesium* was recently demonstrated to be rich in species [5], and examples of hyperdiversity are highly elaborate in the other eutardigrade order, Parachela, which contains such intensively studied genera as *Macrobiotus*, *Ramazzottius*, *Richtersius*, *Paramacrobiotus* and *Platicrista* [6–11]. Resolving many taxonomic uncertainties not only enhanced overall tardigrade classification [12] by narrowing the extent of intraspecific variability, which was traditionally considered sizeable [13, 14], but also contributed, for example, to the recognition of *Hypsibius exemplaris* as a new model metazoan species [15] that is crucial in research on animal development and cryptobiosis (e.g., [16, 17]). As a consequence of the collaborative endeavors of tardigradologists, tens of new species are described every year [18–22].

Comprising more than a hundred species, *Echiniscus* competes with *Macrobiotus* for the position of the most speciose tardigrade genus [23]. High taxonomic importance was typically assigned to dorsal sculpturing in the *Echiniscus* lineage [24]; recently, following the recognition that the phylogenetic position appeared congruent with morphological data, new monophyletic genera were separated from *Echiniscus* [25, 26]. One of these genera, *Nebularmis*, was created for the widespread Palearctic species *Nebularmis reticulatus* [27, 28] and its kin. These echiniscids exhibit unique dorsal sculpturing that consists of a sponge-like intracuticular layer and large, circular or slightly hexagonal, flat epicuticular granules that may merge, giving the plates an unsculptured appearance, a morphological state unknown in the remainder of the family. Moreover, *Nebularmis* has large red eyes and a highly altered ventral cuticular system, with uniform wrinkling in the central part of the venter and characteristic, clearly delineated, thick and densely granulated subcephalic and genital plates. Such a combination of traits makes *Nebularmis* easy to recognize; nevertheless, the state of taxonomy within the genus leaves much to be desired. Almost all known *Nebularmis* species are dubious, most likely synonymous with *N. reticulatus* [28], and some other potentially valid species, such as the Neotropical *Nebularmis phocae* [29], cannot be identified without redescriptions, as their descriptions are too general. Poor species descriptions continue to be a serious problem when linking the traditionally morphologically based Linnean system of classification with the proliferating use of integrative taxonomic methods [30], posing difficulties that threaten the stability and reliability of tardigrade systematics [31].

With the opportunity to analyse many Indomalayan populations representing this uncommon genus, we aim to present the intrageneric diversity and phylogeography of *Nebularmis* in a new light. Four new species are described from regions that were not previously subjected to tardigrade sampling [32], illustrating that Southeast Asia may be an important source of diversity in this phylum and attain similar status to other established biodiversity hotspots [33]. We amend the definition of *Nebularmis* based on new morphological data, present hypotheses on its evolution, and finally discuss our findings in the context of phylogeny of echiniscids, one of the stunningly morphologically complex tardigrade groups [34]. Last, we appeal for doubling the effort in creating a list of available names in Tardigrada ahead of the forthcoming 15^th^ International Symposium on Tardigrada, as has been successfully undertaken for another meiofaunal phylum, Rotifera [35], to officially abandon the names of tens of unidentifiable species.

## Materials and methods

### Sampling, data collection, comparative material and terminology

Individuals belonging to the rare genus *Nebularmis* were extracted from 11 moss and lichen samples collected in South and Southeast Asia (details in Table 1). Samples were air-dried, sealed in paper envelopes, subsequently rehydrated using tap water, and vigorously shaken in beakers; then, the supernatant was transferred to measuring cylinders. After removal of excess water, sediments containing bryophilous and lichenophilous animals and plant particles were poured onto Petri dishes, and tardigrades were detected using stereomicroscopes. Each specimen was drawn into a glass pipette and placed in distilled water. After extraction, the animals were divided into three groups to be used in different analyses: (I) qualitative and quantitative morphological investigations with light contrast microscopy (LCM), specifically phase contrast (PCM) and Nomarski differential interference contrast microscopy (NCM); (II) qualitative morphological observation with scanning electron microscopy (SEM); and (III) DNA sequencing analysis. Specimens of *Nebularmis* spp. inhabiting the Ruwenzori Mountains (1 individual; 2520 m asl, granite stones at the edge of a stream; vicinity of Mubuku, Uganda, Africa; leg. J. Michejda, February 1974), Llanganuco Valley (1 individual; 2400 m asl, granite rocks; Cordillera Blanca, the Andes, Peru, South America; leg. L. Wilczyński, August 1973), Doi Inthanon (ZMUC-516, 519–20; 4 individuals; Thailand, Asia; leg. H. Enghoff, October 1981), and Greenland ([36], currently deposited in Copenhagen) were used for comparative purposes. Data from Gąsiorek et al. [28] were also utilized. Additionally, we examined specimens of *Echiniscus palmai* (NZ-381; 1 individual; ca. 560 m asl; the Haast Pass (Tioripatea), Mount Aspiring National Park, the South Island of New Zealand; leg. D.S. Horning, October 1970; NZ-525; 1 individual; Canaan Road, Abel Tasman National Park, the South Island of New Zealand; leg. D.S. Horning, April 1971).

**Table 1:** List of populations used in the analyses. Types of analyses: (LCM) imaging and morphometry using PCM/NCM, (SEM) imaging using SEM, (DNA) DNA sequencing. The number in each analysis indicates how many specimens were analyzed by a given method (a – adults, v – exuvia, j – juveniles, l – larvae)

Species	Sample code	Coordinates and altitude	Locality	Sample type	Collector and collection date	Analyses		
						LCM	SEM	DNA
*Nebularmis auratus* **sp. nov.**	MM.003	17°28′55″N 97°05′53″E 1073 m asl	Myanmar, Mon, Eastern Yoma Mountains, Kyaiktiyo	moss from tree bark	Dominika Wilkosz; 02.02.2016	3a + 2j	–	4a
*Nebularmis bhutanensis* **sp. nov.**	BT.001	37°29′32″N 89°21′49″E 3120 m asl	Bhutan, Eastern Himalayas, Paro Taktsang	moss from rock	Cristina Cruz & Jorge Domingos; 28.08.2018	3a	–	–
*Nebularmis burmensis* **sp. nov.**	MM.010	20°38′28″N 97°04′14″E 1333 m asl	Myanmar, Shan, Shan Hills, Taunggyi, Kakku Pagodas	moss and lichen from tree bark	Katarzyna Vončina; 21.02.2019	7a + 1j + 1 l	3a	4a
*Nebularmis cirinoi*	ID.517	1°51′20″S 120°19′25″E 1331 m asl	Indonesia, Celebes, Sulawesi Tengah, Lore Lindu, Bada Lembah	moss from tree bark	Piotr Gąsiorek & Artur Oczkowski; 25.08.2017	10a + 4j	3a	2a
	ID.518	1°51′20″S 120°19′30″E 1311 m asl	Indonesia, Celebes, Sulawesi Tengah, Lore Lindu, Bada Lembah	moss and lichen from tree bark	Piotr Gąsiorek & Artur Oczkowski; 25.08.2017	2a	–	–
	ID.874	0°39′55″N 127°24′38″E 1211 m asl	Indonesia, Maluku Utara, Tidore, Gunung Kiematubu	moss and lichen from tree bark	Piotr Gąsiorek; 04.07.2018	1a	–	–
	ID.882	0°40′07″N 127°24′53″E 905 m asl	Indonesia, Maluku Utara, Tidore, Gunung Kiematubu	moss from tree bark	Piotr Gąsiorek; 04.07.2018	1a	–	–
*Nebularmis indicus* **sp. nov.**	IN.040	15°05′44″N 74°12′41″E 77 m asl	India, Goa, Western Ghats, Netravali	moss from tree bark	Joanna Ciosek; 16.09.2019	3a	–	1a
	IN.041	15°03′49″N 74°14′17″E 328 m asl	India, Goa, Western Ghats, Netravali	moss from concrete wall	Joanna Ciosek; 16.09.2019	1a	–	–
	IN.075	14°58′01″N 74°09′30″E 100 m asl	India, Goa, Western Ghats, Cotigao	moss from tree bark in forest canopy	Joanna Ciosek; 14.09.2019	1j	2a	1v with eggs
	IN.076	14°58′01″N 74°09′30″E 100 m asl	India, Goa, Western Ghats, Cotigao	moss from tree bark in forest canopy	Joanna Ciosek; 14.09.2019	1 l	–	1a

The terminology for sclerotized structures follows that by Kristensen [34]. The division of a cephalic cirrus into cirrophore and flagellum is in accordance with Møbjerg et al. [37]. Isonych spurs share identical morphology on all claws, whereas heteronych spurs signify that the spurs on claw IV are different in size/shape/position on the claw branch than the spurs on claws I–III. Abbreviations used for scientific institutions are as follows: UJ – Jagiellonian University (Poland), UP – University of Porto (Portugal). The publication was registered in ZooBank under the following: urn:lsid:zoobank.org:pub:162A7916-4D32-4BA3-9542-624D702BFD16.

### Microscopy, imaging and morphometry

Specimens subjected to light microscopy and morphometry analyses were mounted in a small drop of Hoyer’s medium and examined under an Olympus BX 51 PCM and NCM paired with an Olympus DP74 digital camera. Specimens for SEM imaging were CO_2_ critical point-dried, coated with gold and examined with a Versa 3D DualBeam SEM at the ATOMIN facility of Jagiellonian University. All figures were assembled in Corel Photo-Paint X8. For deep structures that could not be fully focused in a single LCM photograph, a series of images were taken at approximately every 0.1 mm of vertical focusing and then assembled manually in Corel Photo-Paint into a single deep-focus image. All measurements were performed using PCM. Structures were measured only when oriented properly and not broken or deformed. Body length was measured from the anterior to the posterior end of the body, excluding the hind legs. The *sp* index is the ratio of the length of a given structure to the length of the scapular plate [38]. Morphometric data were handled using the Echiniscoidea ver. 1.3 template available from the Tardigrada Register, http://tardigrada.net/register [39]. Raw morphometric data for the new species were deposited in the Tardigrada Register under the following numbers: 0076 (*N. auratus* **sp. nov.**), 0077 (*N. bhutanensis* **sp. nov.**), 0078 (*N. burmensis* **sp. nov.**), and 0079 (*N. indicus* **sp. nov.**). DNA sequences were deposited in GenBank.

### Genotyping and preliminary work on sequences

DNA was extracted from individual animals following a Chelex® 100 resin (Bio-Rad) extraction method [40, 41]. Vouchers were obtained after extraction when possible [42]. Five DNA fragments were sequenced: the 18S small ribosomal rRNA subunit, the 28S large ribosomal rRNA subunit, internal transcribed spacers ITS-1 and ITS-2, and the cytochrome oxidase I (COI) subunit. All fragments were amplified and sequenced according to the protocols described in Stec et al. [41]; primers and original references for specific PCR programs are listed in Supplementary Material 1. GenBank accession numbers for all species are provided in Table 2. 18S rRNA, 28S rRNA, and ITS sequences were aligned with sequences from *Echiniscus testudo* [43] and *Diploechiniscus oihonnae* [44] as outgroups using the Q-INS-i strategy in MAFFT version 7 [45, 46]. The aligned fragments were edited and checked manually in BioEdit [47], with gaps left intact. COI was aligned with the ClustalW Multiple Alignment tool [48] in BioEdit, and uncorrected pairwise distances were calculated using MEGA7 [49]. Alignments are provided in Supplementary Materials 2, 3, 4, 5 and 6.

**Table 2:** GenBank accession numbers for the *Nebularmis* spp. analyzed in this work, bold font indicates new sequences

Species	18S rRNA	28S rRNA	ITS-1	ITS-2	COI
*Nebularmis auratus* **sp. nov.**	**MW180881**	**MW180904**	**MW180893**	**MW180889**	**MW178237**
*Nebularmis burmensis* **sp. nov.**	**MW180882–3**	**MW180905–6**	**MW180894–5**	**MW180890–1**	**MW178238**
*Nebularmis cirinoi*	MK529692, **MW180884**	MK529722, **MW180907**	–	MN271705, **MW180892**	–
*Nebularmis indicus* **sp. nov.**	**MW180885–6**	**MW180908–9**	**MW180896–7**	–	**MW178239–41**
*Nebularmis reticulatus*	MK529693	MK529723	MN271708	MN271700	MN263917–8

### Phylogenetic and biogeographic analyses

The sequences for the nuclear gene fragments were concatenated to generate a 2803-bp matrix in SequenceMatrix ([50]; see Supplementary Material 7). Using PartitionFinder version 2.1.1 [51], with the applied Bayesian information criterion (BIC) and greedy algorithm [52], the best substitution model and partitioning scheme were chosen for posterior phylogenetic analysis. As the best-fit partitioning scheme, PartitionFinder suggested two partitions (I: 18S rRNA +28S rRNA, II: ITS-1 + ITS-2), and the best-fit model was GTR + I + G for both partitions. To obtain a set of Bayesian phylogenetic trees needed for the biogeographic analyses, the original matrix was analyzed using BEAST [53]. Four combinations of clock and tree priors were chosen and run in parallel: (a) a random local clock [54] with the coalescent tree prior, (b) a random local clock with speciation: Yule process as the tree prior, (c) a strict clock [55] with the coalescent tree prior, and (d) a strict clock with speciation: Yule process as the tree prior. Tree searches were run for 10 million generations, sampling the tree every 1000 steps. The trees were summarized with TREEANNOTATOR software (distributed with BEAST), with the first 1000 trees removed. Tracer v1.3 [56] was then used to ensure that Markov chains had reached stationarity and to determine the correct ‘burn-in’ for the analysis, i.e., the first 10% of generations. The effective sample size values were greater than 200, and consensus trees were obtained after summarizing the resulting topologies and discarding the ‘burn-in’ data. All final consensus trees were viewed and visualized by FigTree v.1.4.3, available from https://tree.bio.ed.ac.uk/software/figtree.

Consensus trees constructed from datasets a and c–d shared identical topologies, whereas the tree based on dataset b was divergent from the remaining phylogenies. Consequently the first 9000 trees were removed from the set of trees a and b, and the remaining 1000 trees were used in independent statistical dispersal-vicariance analyses (S-DIVA) [57, 58], implemented in RASP [59], 2020) with phylogenetic uncertainty considered in the calculations. *Nebularmis* species distributions were coded as broad but regionalized (e.g., old historical records of *N. reticulatus* outside of the Palearctic realm were discarded as being unreliable and most likely representing other *Nebularmis* species). The maximum number of areas at a node was set to 3.

## Results

### Taxonomic account

Phylum: Tardigrada Doyère, 1840 [43].

Class: Heterotardigrada Marcus, 1927 [60].

Order: Echiniscoidea Richters, 1926 [61].

Family: Echiniscidae Thulin, 1928 [62].

Genus: *Nebularmis* Gąsiorek & Michalczyk, 2020 in Gąsiorek et al. [26]

Amended diagnosis: Small- to medium-sized echiniscids with red granulate eyes. Long, rigid and thick buccal tube lacking stylet supports. Cirrophores of the cephalic cirri weakly outlined. Only cephalic cirri present. Two pairs of segmental plates and three median plates. Incisions (notches) on caudal plate. Pseudosegmental plates absent. Dorsal plate sculpture composed of an intracuticular sponge layer and large epicuticular, round or slightly hexagonal flat granules that may be connected by *striae*. Additionally, occasional sparse intracuticular pillars and/or micropores may be visible in some portions of the dorsal plates, especially in the scapular plate. Ventral plates present, developed as subcephalic and genital plates. Ventral cuticle wrinkled. Long, sabre-like claws. Larvae and juveniles with minute sparse pores and densely arranged intracuticular pillars in the dorsal plates.

### Species: *Nebularmis auratus***sp. nov. **Gąsiorek & Michalczyk

#### ZooBank LSID: 5CD18D26-F072-4BD2-B221-43E0DDE266B3

**Fig. 1:**
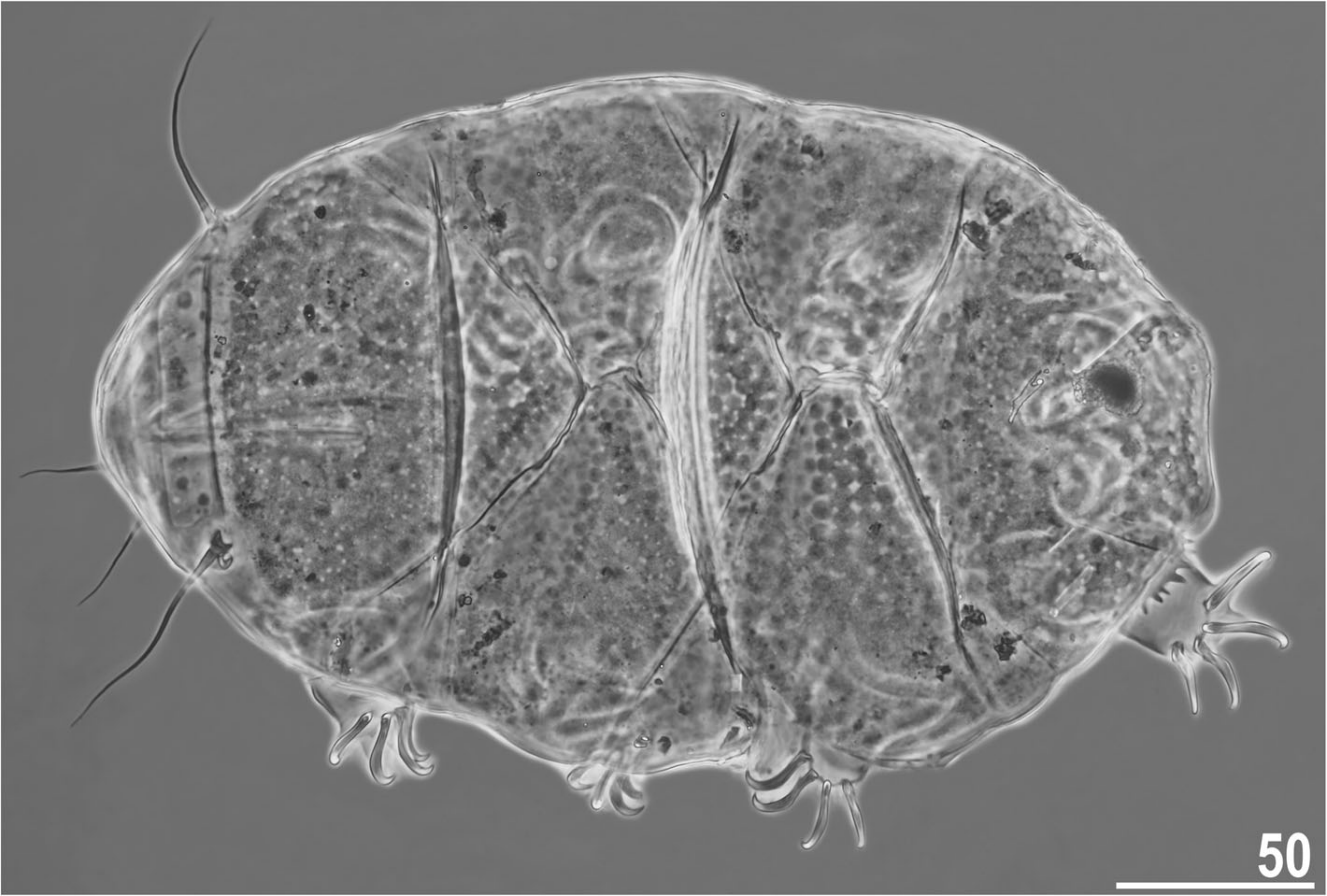
Habitus of *Nebularmis auratus* **sp. nov. **(holotypic ♀ in dorsal view, PCM). Scale bar in μm

**Fig. 2:**
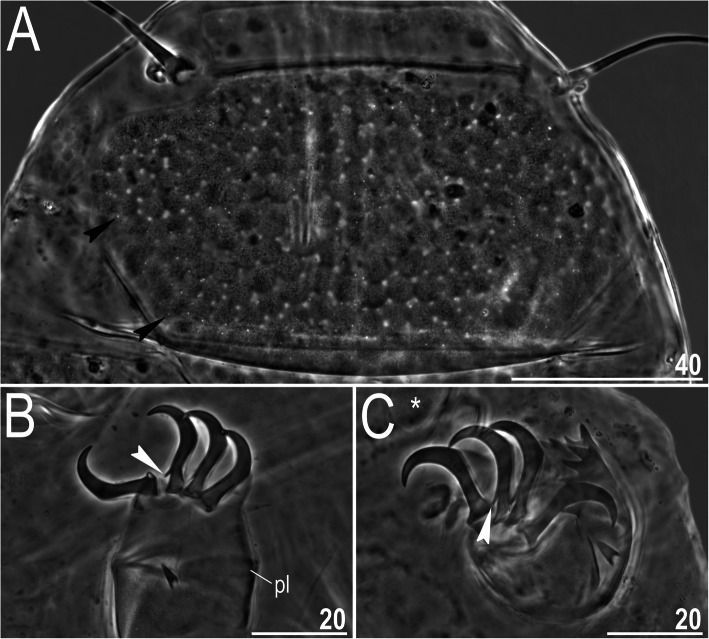
Morphological details of *Nebularmis auratus* **sp. nov.** (PCM): A – scapular plate (note the irregularly distributed micropores), B – claws I, C – claws IV. White arrowheads indicate characteristically blunt-ended primary spurs, black arrowheads – micropores, and asterisk – the genital plate, pl – pulvinus. Scale bars in μm

Figures 1, 2, Table 3.

**Table 3:** Measurements [in μm] of selected morphological structures of adult females and juveniles of *N. auratus* **sp. nov.** (type series) mounted in Hoyer’s medium. Abbreviations: *sp* – the proportion between the length of a given structure and the length of the scapular plate, ? – unknown

CHARACTERISTIC	Holotype (♀)		♀		Juvenile 1		Juvenile 2	
	μm	***sp***	μm	***sp***	μm	***sp***	μm	***sp***
Body length	283	*429*	251	*413*	160	*418*	150	*419*
Scapular plate length	66.0	*–*	60.8	*–*	38.3	*–*	35.8	*–*
Head appendage lengths								
Cirrus *internus*	22.0	*33.3*	18.0	*29.6*	9.4	*24.5*	?	*?*
Cephalic papilla	10.4	*15.8*	9.0	*14.8*	6.7	*17.5*	5.3	*14.8*
Cirrus *externus*	30.0	*45.5*	25.2	*41.4*	17.5	*45.7*	13.4	*37.4*
Clava	6.4	*9.7*	8.2	*13.5*	6.5	*17.0*	?	*?*
Cirrus *A*	59.3	*89.8*	54.9	*90.3*	?	*?*	25.4	*70.9*
Cirrus *A*/body length ratio	21%	*–*	22%	*–*	?	*–*	17%	*–*
Body appendage lengths								
Spine on leg I length	5.7	*8.6*	4.0	*6.6*	3.8	*9.9*	3.1	*8.7*
Papilla on leg IV length	6.2	*9.4*	5.9	*9.7*	4.7	*12.3*	4.5	*12.6*
Number of teeth on the collar	9	*–*	7	*–*	7	*–*	8	*–*
Claw I heights								
Branch	18.6	*28.2*	15.7	*25.8*	11.3	*29.5*	9.6	*26.8*
Spur	3.1	*4.7*	3.0	*4.9*	1.6	*4.2*	2.0	*5.6*
Spur/branch height ratio	17%	*–*	19%	*–*	14%	*–*	21%	*–*
Claw II heights								
Branch	17.0	*25.8*	15.3	*25.2*	10.2	*26.6*	8.6	*24.0*
Spur	?	*?*	2.3	*3.8*	1.6	*4.2*	1.8	*5.0*
Spur/branch height ratio	?	*–*	15%	*–*	16%	*–*	21%	*–*
Claw III heights								
Branch	17.2	*26.1*	14.9	*24.5*	9.8	*25.6*	8.3	*23.2*
Spur	2.7	*4.1*	2.3	*3.8*	1.6	*4.2*	1.7	*4.7*
Spur/branch height ratio	16%	*–*	15%	*–*	16%	*–*	20%	*–*
Claw IV heights								
Branch	22.2	*33.6*	19.2	*31.6*	12.4	*32.4*	10.2	*28.5*
Spur	3.2	*4.8*	?	*?*	2.1	*5.5*	2.0	*5.6*
Spur/branch height ratio	14%	*–*	?	*–*	17%	*–*	20%	*–*

**Description.** Females (i.e., from the third instar onwards): Body dark orange to red and stout (Fig. 1), with large dark red eyes not visible after mounting in Hoyer’s medium. Elongated, dactyloid cephalic papillae (secondary clavae) and reduced (primary) clavae (Fig. 2a). Peribuccal cirri with cirrophores. Cirrus *A* short, with a weakly outlined cirrophore and distinctly thicker flagellum at its proximal end (Fig. 1, 2a).

Dorsal plates thick and well sclerotized, with an evident intracuticular sponge layer and rather tightly arranged flat epicuticular granules (Fig. 1, 2a). Granules are generally well-spaced on the scapular, median and centromedian portions of the paired segmental plates but merge extensively on the lateral portions of paired segmental plates (Fig. 1). The sculpture appears poorly developed and barely discernible under LCM. Cephalic plate small and similar in width to the evident rectangular cervical (neck) plate (Fig. 2a). Scapular plate with randomly distributed micropores. Median plates m1 and m3 unipartite, the latter reduced and weakly delineated from the caudal (terminal) plate. Median plate m2 bipartite, but its anterior portion reduced analogously to m3. Two pairs of large segmental plates are mostly uniform with no transverse belts. Caudal plate large, with short incisions (Fig. 1).

Ventral cuticle with a pair of subcephalic plates and a pair of densely granulated genital plates (Fig. 2c). Venter uniformly wrinkled. Pedal plates I–III absent, pedal plate IV unsculptured, with dentate collar IV (Fig. 2c). Weakly outlined pulvini present on all legs (Fig. 2b). A spine on leg I (Fig. 2b) and an elongated papilla on leg IV are present (Fig. 2c). Claws I–III shorter than claws IV. External claws on all legs spurless (Fig. 2b–c). Internal claws with spurs positioned at approximately 1/4–1/5 of the claw height and divergent from the main branches; spurs usually with blunt distal ends (Fig. 2b–c).

Males: Not found.

Juveniles (i.e., second instar, sexually immature females): Without a gonopore and with a poreless scapular plate. Otherwise, same as adult females.

Larvae and eggs: Not found.

Type material: Holotype (adult ♀, slide MM.003.01) and 4 paratypes (2♀♀, 2 juveniles; slides MM.003.02–5). All slides deposited in UJ.

Type locality: 17°28′55″N, 97°05′53″E, 1073 m asl; Myanmar, Mon, Eastern Yoma Mountains, Kyaiktiyo; moss from tree bark, mountain deciduous forest. This is the first record of tardigrades from Myanmar.

Etymology: From Latin *auratus* = golden. The name refers to *locus typicus*, as Golden Rock is a typical Buddhist pilgrim destination in Myanmar. An adjective in the nominative singular.

Phylogenetic position. The species was inferred as the basal *Nebularmis* lineage in three of the performed analyses (see Fig. 17a for an exemplar tree). In the fourth Bayesian tree (combination c), *N. auratus* **sp. nov.** formed a clade with *N. reticulatus* (Fig. 17b). Both topologies were weakly supported. The *p*-distances in COI ranged between 14.8% (*N. burmensis* **sp. nov.**, MW178238) to 17.0% (*N. reticulatus*, MN263917).

### *Nebularmis bhutanensis***sp. nov.** Veloso, Fontoura & Gąsiorek

#### ZooBank LSID: 106DC9CF-6147-4866-BF6F-552455F3E8A5

**Fig. 3:**
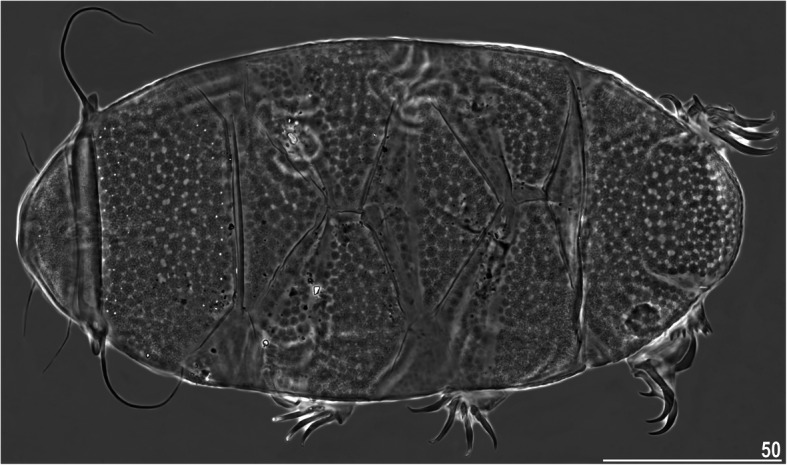
Habitus of *Nebularmis bhutanensis* **sp. nov.** (allotypic ♂ in dorsal view, PCM). Scale bar in μm

**Fig. 4:**
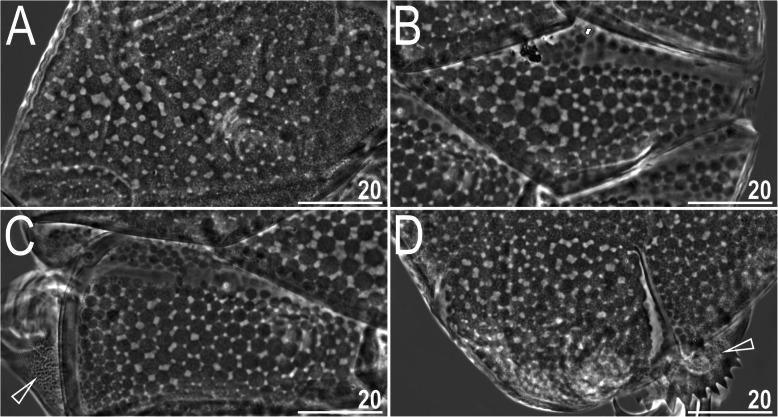
Dorsal sculpturing of *Nebularmis bhutanensis* **sp. nov.** (holotypic ♀, PCM): A – scapular plate, B – median plate II, C – paired segmental plate II, D – caudal (terminal) plate. Arrowheads indicate conspicuous granulation on pedal platelets. Scale bars in μm

**Fig. 5:**
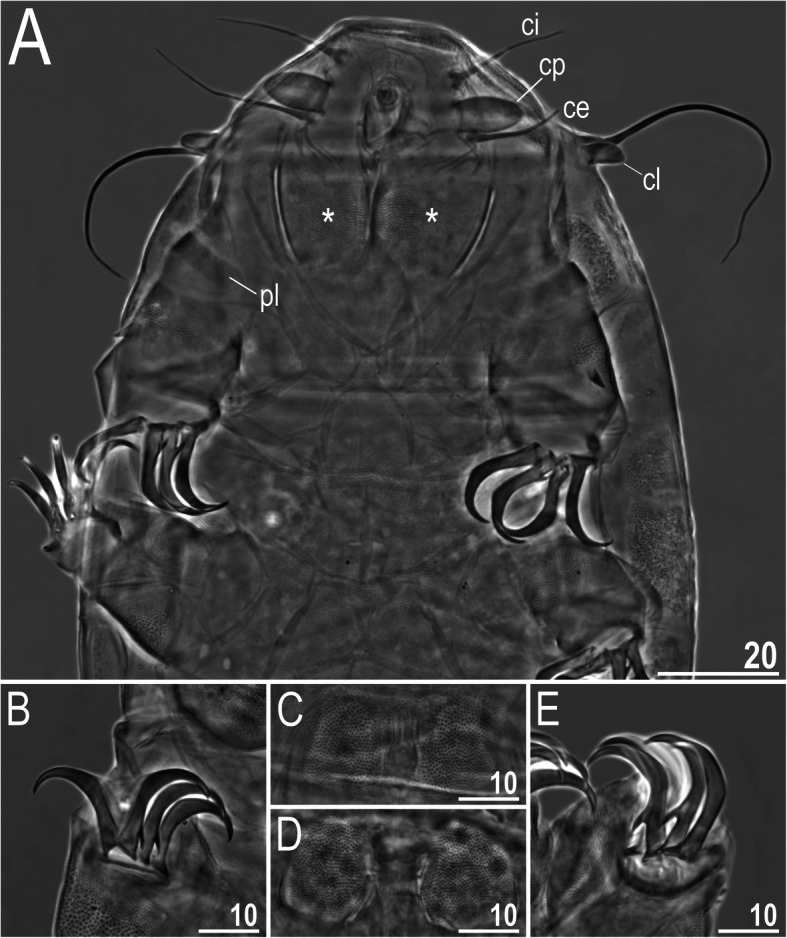
Cephalic, ventral and limb structures of *Nebularmis bhutanensis* **sp. nov.** (PCM): A – anteroventral portion of the body (ce – *cirrus externus*, ci – *cirrus internus*, cl – primary clava, cp – cephalic papilla = secondary clava, pl – pulvinus, * – subcephalic plate; note regular wrinkling, characteristic for the genus), B – claws II, C – genital plates and male gonopore, D – genital plates and female gonopore, E – claws IV. Scale bars in μm

Figures 3, 4, 5, Table 4

**Table 4:** Measurements [in μm] of selected morphological structures of adult *N. bhutanensis* **sp. nov.** (type series) mounted in Hoyer’s medium. Abbreviations: *sp* – the proportion between the length of a given structure and the length of the scapular plate, ? – unknown

CHARACTERISTIC	Holotype (♀)		♂		Allotype (♂)	
	μm	***sp***	μm	***sp***	μm	***sp***
Body length	241^a^	*417*	235^a^	*467*	202	*493*
Scapular plate length	57.8	*–*	50.3	*–*	41.0	*–*
Head appendage lengths						
Cirrus *internus*	16.5	*28.5*	15.9	*31.6*	20.3	*49.5*
Cephalic papilla	10.3	*17.8*	9.8	*19.5*	11.8	*28.8*
Cirrus *externus*	22.6	*39.1*	?	*?*	28.0	*68.3*
Clava	5.4	*9.3*	6.6	*13.1*	7.2	*17.6*
Cirrus *A*	54.9	*95.0*	51.1	*101.6*	52.8	*128.8*
Cirrus *A*/body length ratio	23%	*–*	22%	*–*	26%	*–*
Body appendage lengths						
Spine on leg I length	4.7	*8.1*	3.9	*7.8*	2.9	*7.1*
Papilla on leg IV length	4.2	*7.3*	4.8	*9.5*	5.0	*12.2*
Number of teeth on the collar	10	*–*	11.0	*–*	9	*–*
Claw I heights						
Branch	17.1	*29.6*	15.9	*31.6*	15.5	*37.8*
Spur	3.2	*5.5*	3.1	*6.2*	3.0	*7.3*
Spur/branch height ratio	19%	*–*	19%	*–*	19%	*–*
Claw II heights						
Branch	17.3	*29.9*	15.2	*30.2*	14.5	*35.4*
Spur	3.1	*5.4*	2.8	*5.6*	2.6	*6.3*
Spur/branch height ratio	18%	*–*	18%	*–*	18%	*–*
Claw III heights						
Branch	17.0	*29.4*	15.1	*30.0*	14.7	*35.9*
Spur	3.0	*5.2*	2.4	*4.8*	2.6	*6.3*
Spur/branch height ratio	18%	*–*	16%	*–*	18%	*–*
Claw IV heights						
Branch	20.1	*34.8*	17.3	*34.4*	18.4	*44.9*
Spur	5.2	*9.0*	3.4	*6.8*	4.9	*12.0*
Spur/branch height ratio	26%	*–*	20%	*–*	27%	*–*

^a^Provided approximately, as both specimens were not fully stretched

**Description.** Females (i.e., from the third instar onwards): Body dark orangish-red and stout, with large dark red eyes not visible after mounting in Hoyer’s medium. Large, swollen cephalic papillae (secondary clavae) and bluntly terminated (primary) clavae. Peribuccal cirri with short cirrophores. Cirrus *A* short, with evident cirrophore and slightly thicker flagellum at its proximal end.

Dorsal plates thick and well sclerotized, with an evident intracuticular sponge layer and widely spaced flat epicuticular granules connected by *striae* of various thicknesses (Fig. 4). Granules merge partially in only lateral plate portions (Fig. 4a). Sculpture is obvious under LCM. Cephalic plate small and shorter than the evident rectangular cervical (neck) plate. Scapular plate with micropores distributed along its anterior and posterior edges. Median plates m1 and m3 unipartite, the latter extremely reduced and narrow. Median plate m2 bipartite, with the anterior portion reduced analogously to m3 (Fig. 4b). Two pairs of large segmental plates mostly uniform and with narrow transverse belts delimiting reduced anterior portions (Fig. 4c). Caudal plate large, with short incisions (Fig. 4d).

Ventral cuticle with a pair of subcephalic plates and a pair of densely granulated genital plates (Fig. 5d). Uniform ventral wrinkling present. Pedal plates I–IV sculptured and strongly granulated (Fig. 4c–d), plate IV with a dentate collar (Fig. 4d). Weakly outlined pulvini present on all legs. A spine on leg I and an elongated papilla on leg IV are present. Claws I–III are shorter than claws IV. External claws on all legs spurless (Fig. 5b, e). Internal claws with spurs positioned at approximately 1/4–2/5 of the claw height and heteromorphic, more divergent from branches on claws IV than on claws I–III (Fig. 5e).

Males (i.e., from the third instar onwards): Qualitatively similar to females. Body ovoid (Fig. 3). Cephalic papillae swollen and very large, especially when compared to the (primary) clavae, with blunt ends (Fig. 5a). Gonopore circular, located between the trapezoidal granulated genital plates with a valvate slit separating them (Fig. 5c).

Juveniles, larvae and eggs: Not found.

Type material: Holotype (adult ♀, slide BT.001.01), allotype (adult ♂, slide BT.001.02) and one paratype (adult ♂, slide AS.PE-H33). Holotype and allotype deposited in UJ, paratype deposited in UP.

Type locality: 37°29′32″N, 89°21′49″E, 3120 m asl; Bhutan, Eastern Himalayas, Paro Taktsang; moss from rock, pine forest. The first record of tardigrades from Bhutan.

Etymology: From Latin *bhutanensis* = inhabiting Bhutan. The name underlines *terra typica*. An adjective in the nominative singular.

Phylogenetic position. Unknown. The species has the most distinctive sculpture subtype within the entire genus; thus, acquiring DNA sequences is important from a phylogenetic perspective.

### *Nebularmis burmensis***sp. nov.** Gąsiorek & Vončina

#### ZooBank LSID: FCABDC67-E90D-4BB6-888A-0C518D3C6BC2

**Fig. 6:**
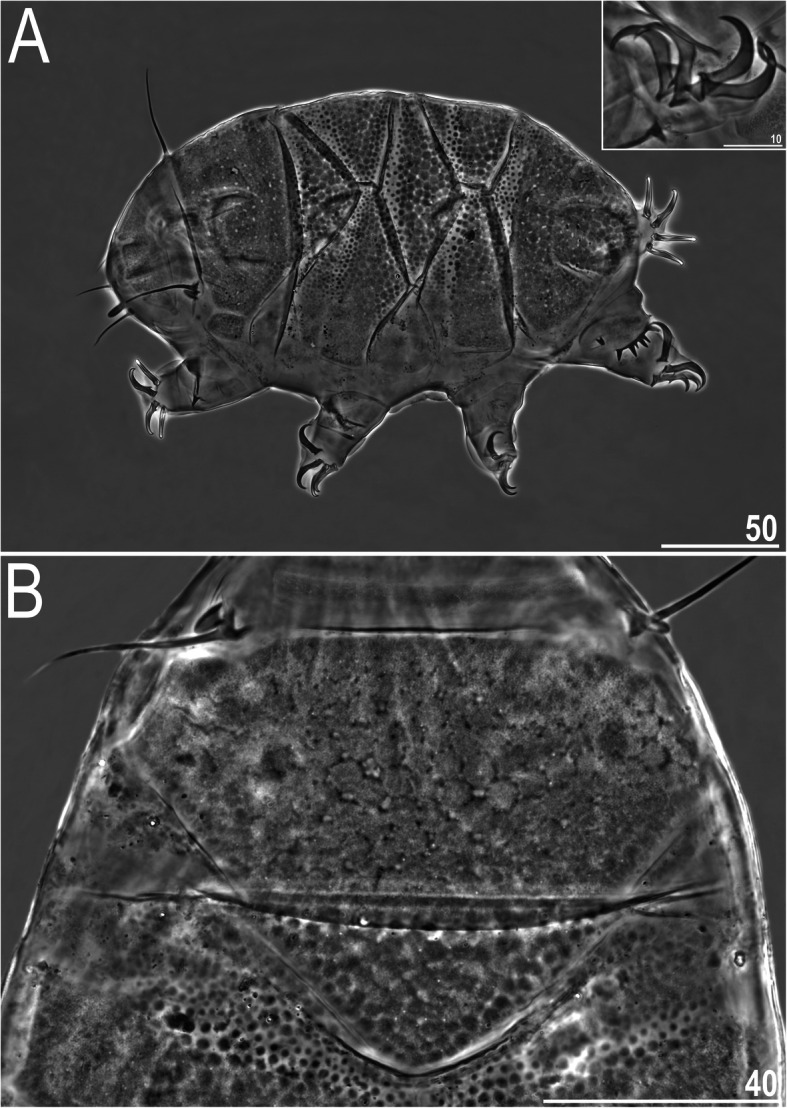
Habitus of *Nebularmis burmensis* **sp. nov. **(PCM): A – holotypic ♀ in dorsolateral view (insert shows claws I), B – dorsal sculpturing of the anteriormost portion of the body. Scale bars in μm

**Fig. 7:**
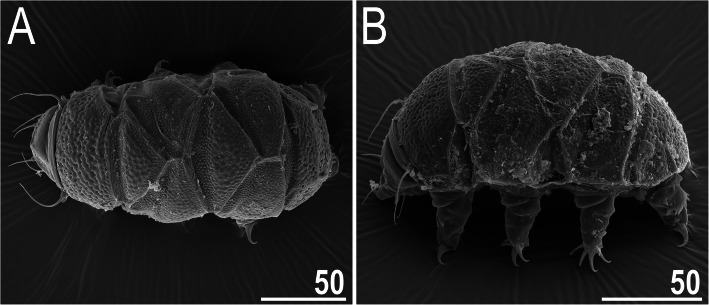
Habitus of *Nebularmis burmensis* **sp. nov.** (SEM): A – dorsal view, B – dorsolateral view. Scale bars in μm

**Fig. 8:**
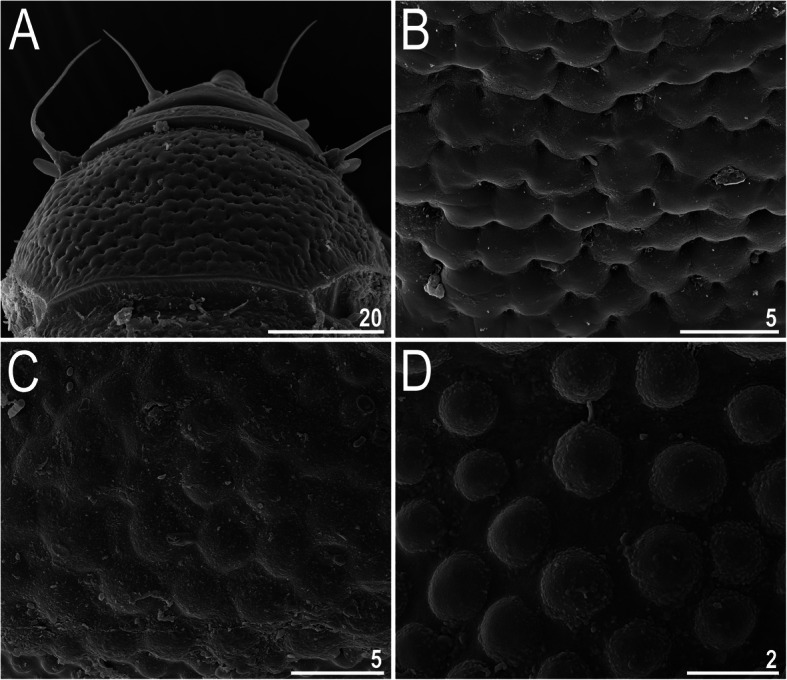
Morphological details of *Nebularmis burmensis* **sp. nov.** (SEM): A – head, B – central portion of the scapular plate, C – central portion of the median plate I, D – epicuticular bumps covering the anterior portions of median and paired segmental plates in close-up. Scale bars in μm

Figures 6, 7, 8, Table 5.

**Table 5:** Measurements [in μm] of selected morphological structures of adult female *N. burmensis* **sp. nov.** (type series) mounted in Hoyer’s medium. Abbreviations: N – number of specimens/structures measured, RANGE refers to the smallest and the largest structure among all the measured specimens; SD – standard deviation; *sp* – the proportion between the length of a given structure and the length of the scapular plate

CHARACTERISTIC	N	RANGE						MEAN		SD		Holotype	
		μm			***sp***			μm	***sp***	μm	***sp***	μm	***sp***
Body length	7	217	–	236	*407*	*–*	*465*	227	*432*	6	*21*	227	*440*
Scapular plate length	7	49.6	–	55.6		–		52.6	*–*	2.2	*–*	51.6	*–*
Head appendage lengths													
Cirrus *internus*	7	15.3	–	18.5	*30.6*	*–*	*34.8*	17.3	*32.8*	1.4	*1.7*	17.0	*32.9*
Cephalic papilla	7	6.8	–	8.6	*13.0*	*–*	*16.8*	7.9	*15.1*	0.6	*1.1*	8.0	*15.5*
Cirrus *externus*	7	20.6	–	25.0	*38.7*	*–*	*47.1*	22.8	*43.4*	1.7	*3.2*	23.2	*45.0*
Clava	7	5.9	–	7.8	*11.8*	*–*	*14.0*	6.8	*12.9*	0.6	*0.8*	7.1	*13.8*
Cirrus *A*	7	36.8	–	43.5	*70.1*	*–*	*79.6*	39.6	*75.3*	2.1	*3.3*	38.9	*75.4*
Cirrus *A*/body length ratio	7	16%	–	19%		–		17%	*–*	1%	*–*	17%	*–*
Body appendage lengths													
Spine on leg I length	7	4.0	–	5.0	*7.9*	*–*	*9.4*	4.5	*8.6*	0.3	*0.6*	4.7	*9.1*
Papilla on leg IV length	7	4.0	–	4.9	*8.0*	*–*	*9.5*	4.6	*8.7*	0.3	*0.6*	4.7	*9.1*
Number of teeth on the collar	7	8	–	10		–		9.0	*–*	0.6	*–*	10	*–*
Claw I heights													
Branch	7	14.6	–	16.5	*26.6*	*–*	*29.8*	15.0	*28.5*	0.7	*1.2*	14.6	*28.3*
Spur	7	1.9	–	2.6	*3.4*	*–*	*5.0*	2.2	*4.2*	0.3	*0.5*	2.2	*4.3*
Spur/branch height ratio	7	13%	–	18%		–		15%	*–*	2%	*–*	15%	*–*
Claw II heights													
Branch	7	14.1	–	15.9	*25.7*	*–*	*29.2*	14.6	*27.7*	0.6	*1.2*	14.2	*27.5*
Spur	6	1.8	–	2.1	*3.3*	*–*	*4.1*	2.0	*3.8*	0.1	*0.3*	2.1	*4.1*
Spur/branch height ratio	6	13%	–	15%		–		14%	*–*	1%	*–*	15%	*–*
Claw III heights													
Branch	7	13.8	–	16.4	*26.0*	*–*	*31.0*	15.0	*28.5*	1.0	*1.8*	15.0	*29.1*
Spur	5	1.8	–	2.7	*3.4*	*–*	*4.9*	2.1	*3.9*	0.4	*0.6*	2.0	*3.9*
Spur/branch height ratio	5	12%	–	16%		–		14%	*–*	2%	*–*	13%	*–*
Claw IV heights													
Branch	7	18.1	–	20.5	*32.9*	*–*	*37.1*	18.6	*35.5*	0.9	*1.6*	18.6	*36.0*
Spur	3	2.7	–	3.0	*5.1*	*–*	*5.8*	2.9	*5.4*	0.2	*0.4*	3.0	*5.8*
Spur/branch height ratio	3	15%	–	16%		*–*		16%	*–*	1%	*–*	16%	*–*

**Description.** Females (i.e., from the third instar onwards): Body dark orange and stout (Fig. 6a, 7), with large dark red eyes not visible after mounting in Hoyer’s medium. Elongated, dactyloid cephalic papillae (secondary clavae) and reduced (primary) clavae (Fig. 6, 8a). Peribuccal cirri with cirrophores. Cirrus *A* short, with a weakly defined cirrophore and smooth proximal end of the flagellum that is approximately equal in width to its distal end (Fig. 6, 8a).

Dorsal plates thick and strongly sclerotized, with a poorly visible intracuticular sponge layer (Fig. 6b). Flat epicuticular granules well-spaced only in the scapular plate (Fig. 6b, 7, 8a–b) and anterior portions of the paired plates, in which the granules become pronounced convex bumps (Fig. 8d). In the remaining portions of the dorsal armor, the sculpture is poorly developed and barely discernible under both LCM and SEM (Fig. 6a, 7, 8c). Cephalic plate is extremely small, with an anterior keel-shaped incision (Fig. 6a); the rectangular cervical (neck) plate is also poorly visible (Fig. 8a). Scapular plate without micropores (Fig. 6b, 8a–b). Median plates m1 and m3 unipartite, the latter rudimentary and weakly delineated from the caudal (terminal) plate (Fig. 6a, 7a). Median plate m2 bipartite, with the anterior portion reduced analogously to m3. Two pairs of large segmental plates, with evident transverse belts containing blunt hemispherical granules. Caudal plate large, with short incisions (Fig. 6a, 7b).

Ventral cuticle with a pair of subcephalic plates and a pair of genital plates. Regularly spaced ventral wrinkling present. Pedal plates I–III absent, pedal plate IV unsculptured, with dentate collar IV (Fig. 6a, 7b). Weakly outlined pulvini present on all legs (Fig. 7b). A spine on leg I and an elongated papilla on leg IV are present (Fig. 6a, 7b). Claws I–III shorter than claws IV. External claws on all legs spurless (Fig. 6a, insert). Internal claws with short, divergent spurs positioned at approximately 1/4–1/5 of the claw height.

Males: Not found.

Juveniles (i.e., second instar): Body 139 μm long (scapular plate length 39.5 μm). Cephalic appendage lengths: *cirrus internus* 11.4 μm, cephalic papilla 5.6 μm, *cirrus externus* 12.8 μm, cirrus *A* 23.0 μm. Body appendage lengths: spine I 2.9 μm, papilla IV 3.0 μm. Five teeth on the collar. Claw branch heights: 9.1–10.8 μm (spurs 1.7–1.9 μm). One qualitative difference with respect to adults is the lack of gonopore.

Larvae (i.e., first instar): Body 120 μm long (scapular plate length 28.1 μm). Cephalic appendage lengths: *cirrus internus* 10.0 μm, cephalic papilla 4.6 μm, *cirrus externus* 11.6 μm, (primary) clava 5.0 μm, cirrus *A* 18.9 μm. Body appendage lengths: spine I 1.7 μm, papilla IV 2.6 μm. Five teeth on the collar. Claw branch heights: 7.5–8.7 μm (spurs 1.4–1.9 μm). Gonopore and anus absent.

Eggs: Not found.

Type material: Holotype (adult ♀, slide MM.010.11) and 8 paratypes (6♀♀, 1 juvenile, 1 larva; slides MM.010.08–10, 12–16). Slides MM.010.08–14 deposited in UJ, MM.010.15–16 deposited in UP.

Type locality: 20°38′28″N, 97°04′14″E, 1333 m asl; Myanmar, Shan, Shan Hills, Taunggyi, Kakku Pagodas; moss and lichen from tree bark, rural habitat. The first record of tardigrades from Myanmar.

Etymology: From Latin *burmensis* = inhabiting Burma (the postcolonial name of Myanmar). The name underlines *terra typica*. An adjective in the nominative singular.

Phylogenetic position. The species was inferred to be a sister species to *Nebularmis indicus* **sp. nov.** (Fig. 17). The *p*-distances in COI ranged from 9.5% (*N. indicus* **sp. nov.**, MW178240) to 14.8% (*N. auratus* **sp. nov.**, MW178237).

### *Nebularmis cirinoi* (Binda & Pilato, 1993) [63]

**Fig. 9:**
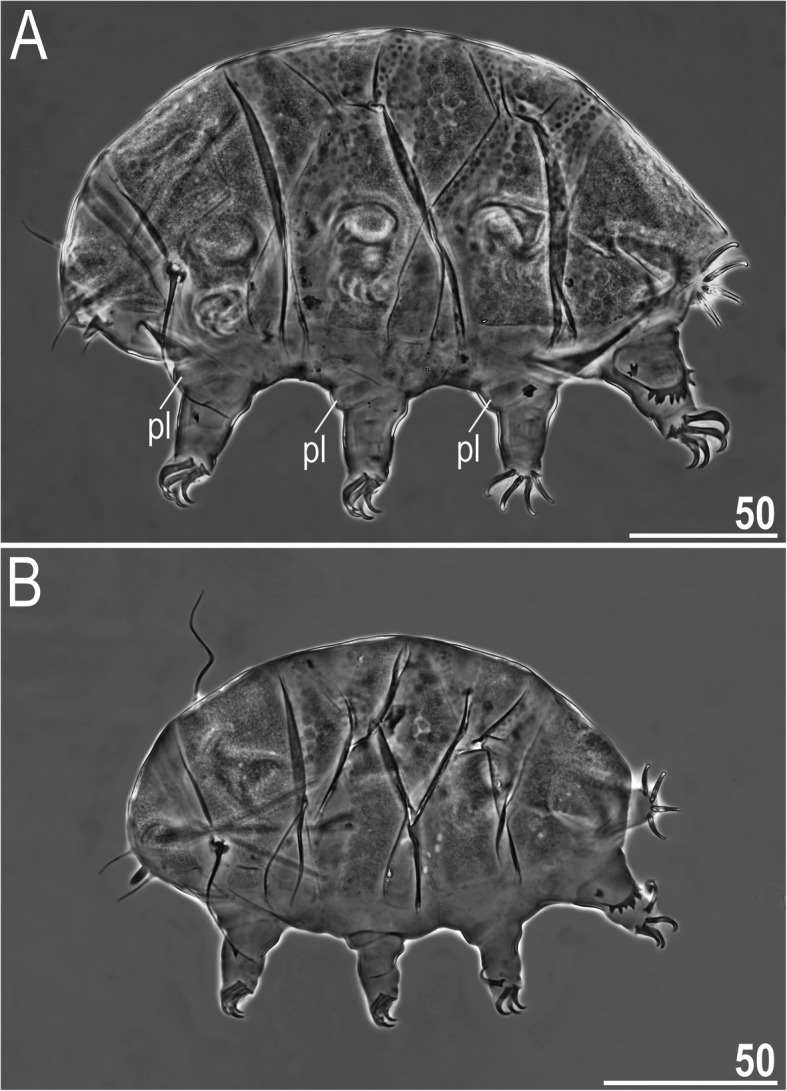
Habitus of *Nebularmis cirinoi* (PCM): A –♀ in dorsolateral view (pl – pulvini), B – juvenile in dorsolateral view. Scale bars in μm

**Fig. 10:**
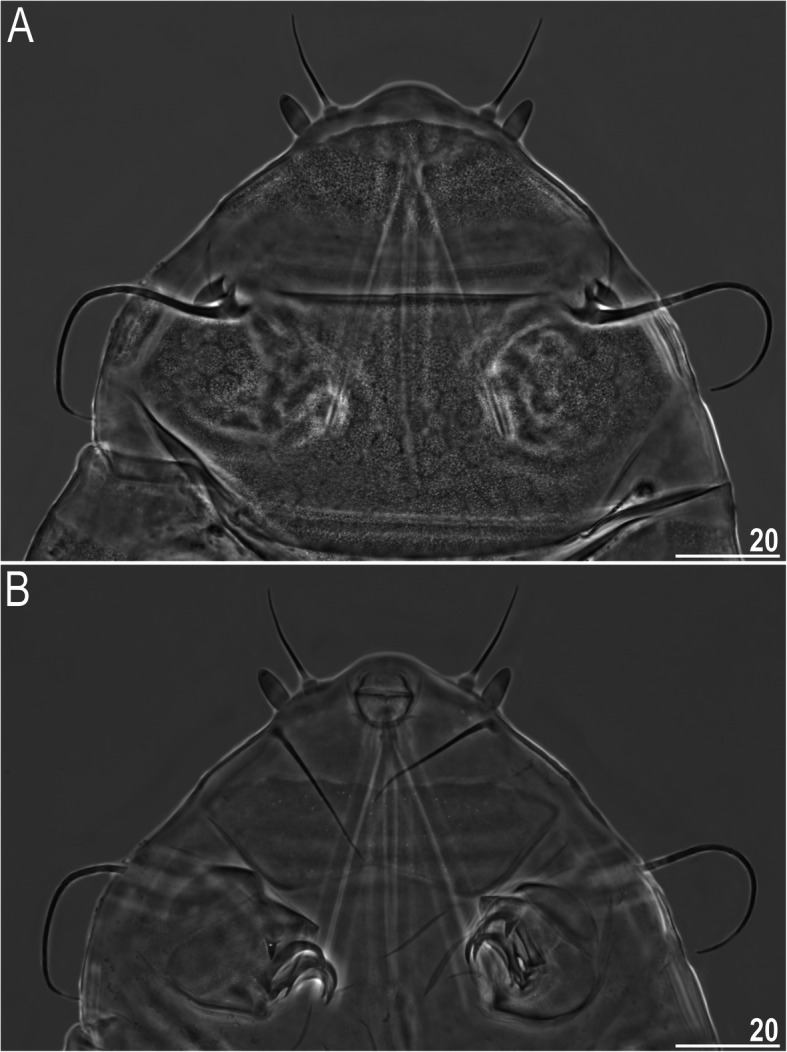
Cephalic and ventral structures of *Nebularmis cirinoi* (PCM): A – anterodorsal portion of the body, B – anteroventral portion of the body (note a pair of large trapezoidal subcephalic plates with irregularly distributed micropores). Scale bars in μm

**Fig. 11:**
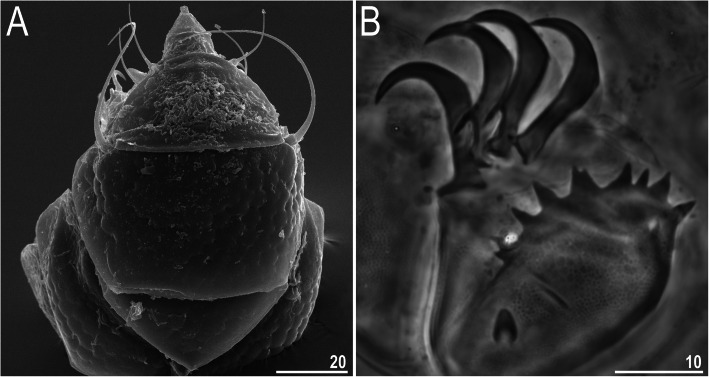
Morphological details of *Nebularmis cirinoi*: A – anterodorsal portion of the body (SEM), B – claws IV. Scale bars in μm

Figures 9, 10, 11, Tables 6 and 7.

**Table 6:** Measurements [in μm] of selected morphological structures of adult females of *N. cirinoi* (Indomalayan specimens) mounted in Hoyer’s medium. Abbreviations: N – number of specimens/structures measured, RANGE refers to the smallest and the largest structure among all measured specimens; SD – standard deviation; *sp* – the proportion between the length of a given structure and the length of the scapular plate; ? – unknown

CHARACTERISTIC	N	RANGE						MEAN		SD	
		μm			***sp***			μm	***sp***	μm	***sp***
Body length	9	200	–	252	*384*	*–*	*446*	220	*416*	18	*22*
Scapular plate length	9	45.7	–	62.5		–		53.1	*–*	4.4	*–*
Head appendage lengths											
Cirrus *internus*	8	17.4	–	25.8	*36.2*	*–*	*47.8*	20.8	*40.0*	2.4	*3.9*
Cephalic papilla	9	8.2	–	9.7	*14.9*	*–*	*19.3*	9.2	*17.4*	0.5	*1.4*
Cirrus *externus*	9	20.1	–	26.7	*39.7*	*–*	*49.4*	23.9	*45.2*	2.1	*3.5*
Clava	9	4.5	–	8.2	*8.3*	*–*	*14.9*	6.9	*13.0*	1.1	*2.2*
Cirrus *A*	7	64.8	–	72.2	*112.8*	*–*	*133.7*	68.1	*125.7*	2.6	*7.0*
Cirrus *A*/Body length ratio	7	28%	–	35%		–		30%	*–*	2%	*–*
Body appendage lengths											
Spine on leg I length	8	2.0	–	4.6	*4.4*	*–*	*7.5*	3.5	*6.6*	0.8	*1.1*
Papilla on leg IV length	8	3.6	–	5.1	*6.2*	*–*	*9.6*	4.3	*8.2*	0.5	*1.0*
Number of teeth on the collar	9	8	–	11		–		9.0	*–*	1.1	*–*
Claw I heights											
Branch	9	12.6	–	15.3	*24.5*	*–*	*28.1*	14.0	*26.5*	0.7	*1.1*
Spur	8	3.1	–	4.1	*5.1*	*–*	*7.6*	3.5	*6.5*	0.4	*0.8*
Spur/branch height ratio	8	21%	–	29%		–		25%	*–*	3%	*–*
Claw II heights											
Branch	9	12.4	–	14.4	*23.0*	*–*	*27.1*	13.3	*25.1*	0.6	*1.3*
Spur	8	2.8	–	4.0	*5.4*	*–*	*6.9*	3.2	*6.0*	0.4	*0.5*
Spur/branch height ratio	8	22%	–	28%		–		24%	*–*	2%	*–*
Claw III heights											
Branch	9	12.3	–	14.3	*22.9*	*–*	*27.8*	13.3	*25.1*	0.6	*1.6*
Spur	9	2.6	–	3.6	*5.0*	*–*	*7.2*	3.2	*6.1*	0.3	*0.8*
Spur/branch height ratio	9	21%	–	27%		–		24%	*–*	2%	*–*
Claw IV heights											
Branch	9	15.2	–	17.4	*26.6*	*–*	*33.7*	16.2	*30.7*	0.7	*2.3*
Spur	1	4.5	–	4.5	*7.2*	*–*	*7.2*	4.5	*7.2*	?	*?*
Spur/branch height ratio	1	27%	–	27%		*–*		27%	*–*	?	*–*

**Table 7:** Measurements [in μm] of selected morphological structures of juveniles of *N. cirinoi* (Indomalayan specimens) mounted in Hoyer’s medium. Abbreviations: N – number of specimens/structures measured, RANGE refers to the smallest and the largest structure among all measured specimens; SD – standard deviation; *sp* – the proportion between the length of a given structure and the length of the scapular plate; ? – unknown

CHARACTERISTIC	N	RANGE						MEAN		SD	
		μm			***sp***			μm	***sp***	μm	***sp***
Body length	5	145	–	192	*374*	*–*	*443*	172	*406*	19	*27*
Scapular plate length	5	35.0	–	51.3		–		42.7	*–*	5.8	*–*
Head appendage lengths											
Cirrus *internus*	5	12.8	–	19.6	*36.6*	*–*	*41.0*	16.5	*38.6*	2.5	*1.7*
Cephalic papilla	5	7.0	–	7.9	*15.2*	*–*	*20.0*	7.5	*17.7*	0.4	*2.0*
Cirrus *externus*	5	14.5	–	21.8	*41.4*	*–*	*48.1*	19.2	*44.9*	2.8	*2.8*
Clava	5	5.1	–	6.9	*12.8*	*–*	*15.3*	6.0	*14.1*	0.7	*1.0*
Cirrus *A*	5	41.2	–	67.0	*117.7*	*–*	*130.6*	54.3	*126.8*	9.2	*5.4*
Cirrus *A*/body length ratio	5	28%	–	35%		–		31%	*–*	3%	*–*
Body appendage lengths											
Spine on leg I length	5	2.6	–	3.4	*6.0*	*–*	*8.1*	2.9	*7.0*	0.3	*1.0*
Papilla on leg IV length	5	2.9	–	5.0	*8.3*	*–*	*9.7*	3.8	*8.9*	0.8	*0.8*
Number of teeth on the collar	5	6	–	9		–		7.6	*–*	1.1	*–*
Claw I heights											
Branch	5	9.2	–	13.8	*26.3*	*–*	*30.1*	11.8	*27.6*	1.7	*1.5*
Spur	5	2.0	–	3.8	*5.7*	*–*	*7.4*	2.9	*6.8*	0.6	*0.7*
Spur/branch height ratio	5	22%	–	28%		–		25%	*–*	3%	*–*
Claw II heights											
Branch	5	9.3	–	12.7	*24.3*	*–*	*26.8*	11.0	*25.8*	1.2	*1.2*
Spur	5	2.1	–	3.5	*5.7*	*–*	*7.3*	2.8	*6.6*	0.5	*0.7*
Spur/branch height ratio	5	23%	–	28%		–		26%	*–*	2%	*–*
Claw III heights											
Branch	5	8.8	–	12.9	*23.1*	*–*	*25.4*	10.6	*24.8*	1.5	*0.9*
Spur	5	2.1	–	3.0	*4.8*	*–*	*7.1*	2.7	*6.3*	0.4	*1.0*
Spur/branch height ratio	5	21%	–	28%		–		25%	*–*	3%	*–*
Claw IV heights											
Branch	5	9.9	–	15.0	*28.3*	*–*	*30.6*	12.7	*29.6*	1.8	*0.9*
Spur	1	3.2	–	3.2	*7.3*	*–*	*7.3*	3.2	*7.3*	?	*?*
Spur/branch height ratio	1	25%	–	25%		*–*		25%	*–*	?	*–*

**Description of Indomalayan populations.** Females (i.e., from the third instar onwards): Body orange and stout (Fig. 9a), with large dark red eyes not visible after mounting in Hoyer’s medium. Elongated, dactyloid cephalic papillae (secondary clavae) and blunt terminated (primary) clavae (Fig. 9a, 10). Peribuccal cirri with bulbous cirrophores (Fig. 10b). Cirrus *A* short to medium in length, with the proximal end of the flagellum smooth and slightly thickened (Fig. 10a).

Dorsal plates thick and well sclerotized, with a dominant visible intracuticular sponge layer (Fig. 10a). Flat, circular epicuticular granules merging on almost all portions of the dorsal plates (Fig. 9a, 10a, 11a). Both cephalic and cervical (neck) plates poorly defined; scapular plate without micropores (Fig. 10a). Median plates m1 and m3 unipartite, the latter rudimentary and weakly delineated from the caudal (terminal) plate (Fig. 9a). Median plate m2 bipartite, with its anterior portion reduced analogously to m3. Two pairs of large segmental plates without transverse belts. Caudal plate of medium size, with short incisions.

Ventral cuticle with a pair of trapezoidal subcephalic plates (Fig. 10b) and a pair of genital plates. Uniform ventral wrinkling present. Pedal plates I–III absent, pedal plate IV weakly sculptured, with dentate collar IV (Fig. 11b). Weakly outlined pulvini present on all legs (Fig. 9a). A small spine on leg I and a conical papilla on leg IV are present (Fig. 9a, 10b, 11b). Claws I–III shorter than claws IV. External claws on all legs smooth. Heteromorphic internal claws with short spurs positioned at approximately 1/3–1/4 of the claw height and more divergent from claw branches on leg IV than those on legs I–III (Fig. 11b).

Juveniles (i.e., second instar): Qualitatively similar to females. Epicuticular granules, with the exception of the central portions of median plates m1–2, completely merged, giving the dorsal plate surface a smooth appearance (Fig. 9b). Gonopore absent.

Larvae and eggs: Not found.

Material examined: Celebes: 12 ♀♀, 4 juveniles (slides ID.517.02, 05, 07, 12, 20–21, 24–28, 32, 37, 37–38, ID.518.05, 20), Tidore: 2♀♀ (slides ID.874.02, ID.882.07). All slides deposited in UJ.

Phylogenetic position. The species was inferred to be a sister taxon to the *Nebularmis burmensis* **sp. nov.** + *Nebularmis indicus* **sp. nov.** clade (Fig. 17).

### *Nebularmis indicus***sp. nov.** Gąsiorek, Ciosek & Michalczyk

#### ZooBank LSID: AEF9CF84-897A-4C08–8383-7E3B440EFA56

**Fig. 12:**
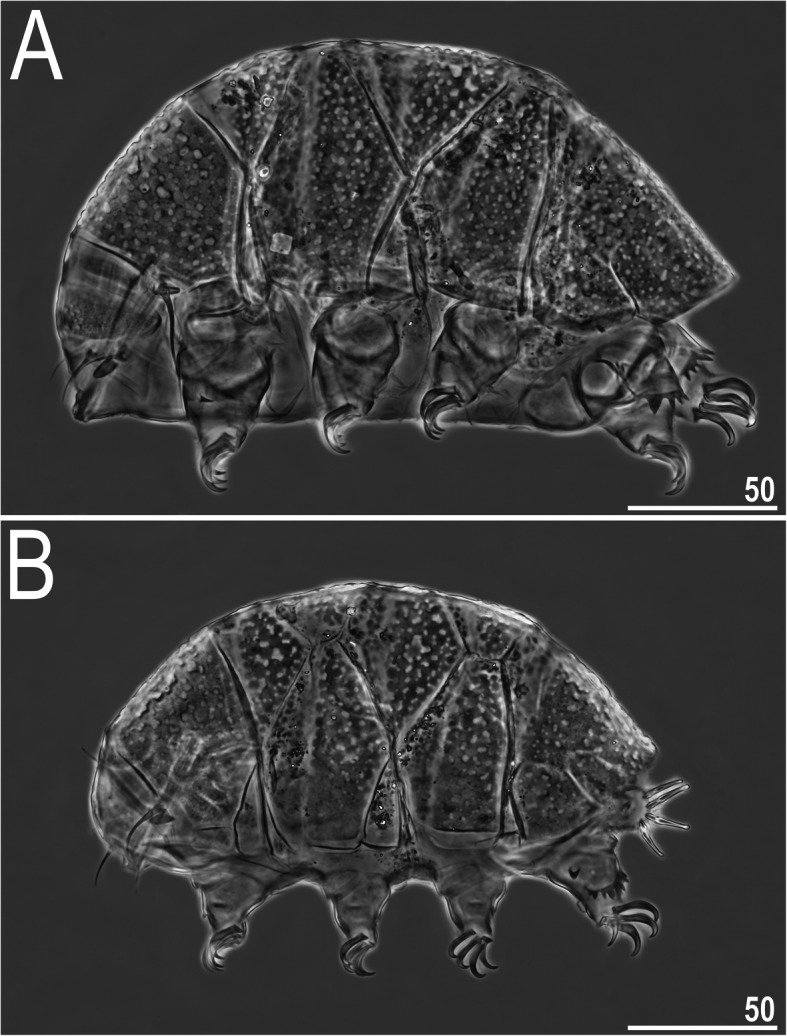
Habitus of *Nebularmis indicus* **sp. nov. **(PCM): A – holotypic ♀ in lateral view, B – allotypic ♂ in dorsolateral view. Scale bars in μm

**Fig. 13:**
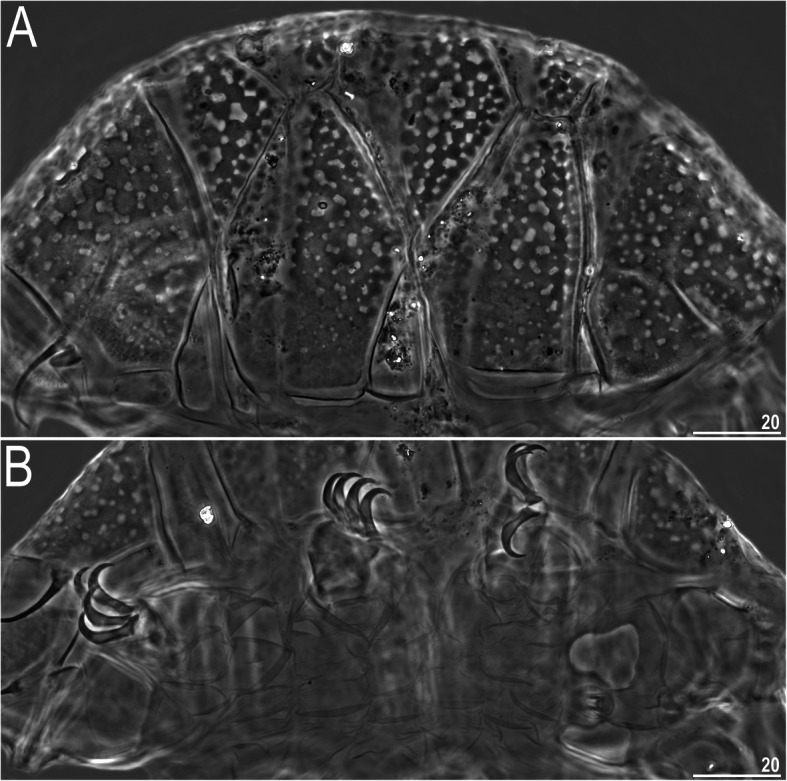
Sculpturing of *Nebularmis indicus* **sp. nov.** (allotypic ♂, PCM): A – dorsal view, B – ventral view (note regular wrinkling characteristic for the genus and male gonopore between smooth genital plates). Scale bars in μm

**Fig. 14:**
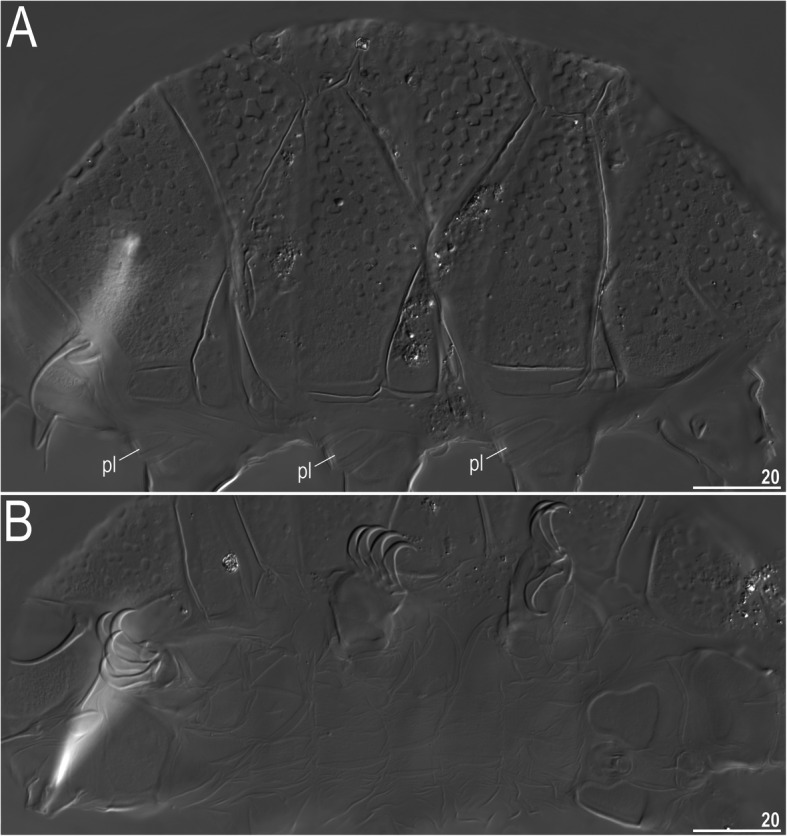
Sculpturing of *Nebularmis indicus* **sp. nov.** (allotypic ♂, NCM): A – dorsal view (pl – pulvini), B – ventral view. Scale bars in μm

**Fig. 15:**
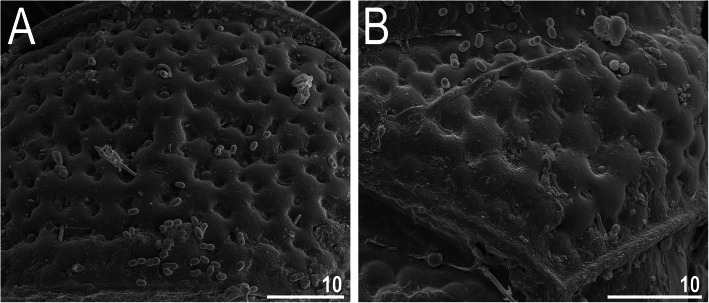
Dorsal sculpturing of *Nebularmis indicus* **sp. nov.** (SEM): A – scapular plate, B – posterior portion of the paired segmental plate II. The anterior directed upwards. Scale bars in μm

**Fig. 16:**
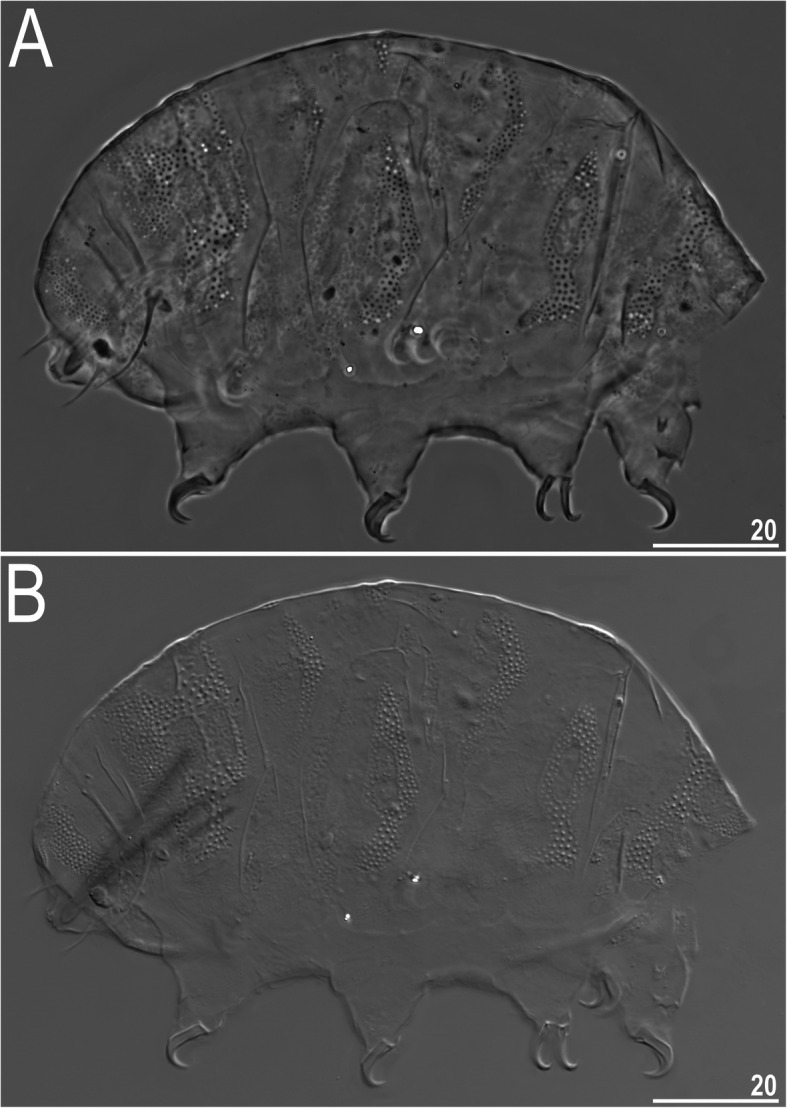
Larval habitus of *Nebularmis indicus* **sp. nov.**: A – dorsolateral view (PCM), B – dorsolateral view (NCM). Scale bars in μm

Figures 12, 13, 14, 15 and 16, Table 8.

**Table 8:** Measurements [in μm] of selected morphological structures of *N. indicus* **sp. nov.** (type series) mounted in Hoyer’s medium. Abbreviations: *sp* – the proportion between the length of a given structure and the length of the scapular plate, ? – unknown

CHARACTERISTIC	Holotype (♀)		♀		Allotype (♂)		♂		Juvenile		Larva	
	μm	***sp***	μm	***sp***	μm	***sp***	μm	***sp***	μm	***sp***	μm	***sp***
Body length	227	*387*	197	*380*	191	*381*	162	*423*	152	*347*	113	*459*
Scapular plate length	58.7	*–*	51.8	*–*	50.1	*–*	38.3	*–*	43.8	*–*	24.6	*–*
Head appendage lengths												
Cirrus *internus*	17.6	*30.0*	14.6	*28.2*	15.9	*31.7*	13.5	*35.2*	11.0	*25.1*	6.6	*26.8*
Cephalic papilla	9.1	*15.5*	7.1	*13.7*	8.5	*17.0*	7.4	*19.3*			3.7	*15.0*
Cirrus *externus*	23.1	*39.4*	18.6	*35.9*	18.2	*36.3*	20.9	*54.6*	15.6	*35.6*	10.6	*43.1*
Clava	7.1	*12.1*	6.8	*13.1*	6.7	*13.4*	7.1	*18.5*	5.9	*13.5*	4.4	*17.9*
Cirrus *A*	48.8	*83.1*	37.0	*71.4*	38.1	*76.0*	42.3	*110.4*	31.1	*71.0*	17.9	*72.8*
Cirrus *A*/body length ratio	21%	*–*	19%	*–*	20%	*–*	26%	*–*	20%	*–*	16%	*–*
Body appendage lengths												
Spine on leg I length	5.2	*8.9*	3.5	*6.8*	3.5	*7.0*	3.9	*10.2*	3.4	*7.8*	1.8	*7.3*
Papilla on leg IV length	5.4	*9.2*	4.6	*8.9*	5.4	*10.8*	4.4	*11.5*	3.2	*7.3*	2.3	*9.3*
Number of teeth on the collar	8	*–*	5	*–*	6	*–*	7	*–*	6	*–*	3	*–*
Claw I heights												
Branch	16.1	*27.4*	14.5	*28.0*	13.0	*25.9*	12.4	*32.4*	11.1	*25.3*	8.0	*32.5*
Spur	2.1	*3.6*	?	*?*	2.4	*4.8*	2.2	*5.7*	2.1	*4.8*	1.8	*7.3*
Spur/branch height ratio	13%	*–*	?	*–*	18%	*–*	18%	*–*	19%	*–*	23%	*–*
Claw II heights												
Branch	14.2	*24.2*	12.6	*24.3*	12.5	*25.0*	11.7	*30.5*	?	*?*	7.3	*29.7*
Spur	2.4	*4.1*	?	*?*	2.2	*4.4*	2.1	*5.5*	?	*?*	1.3	*5.3*
Spur/branch height ratio	17%	*–*	?	*–*	18%	*–*	18%	*–*	?	*–*	18%	*–*
Claw III heights												
Branch	15.1	*25.7*	13.2	*25.5*	12.4	*24.8*	10.6	*27.7*	11.3	*25.8*	7.1	*28.9*
Spur	2.3	*3.9*	1.9	*3.7*	1.8	*3.6*	1.8	*4.7*	?	*?*	1.6	*6.5*
Spur/branch height ratio	15%	*–*	14%	*–*	15%	*–*	17%	*–*	?	*–*	23%	*–*
Claw IV heights												
Branch	18.0	*30.7*	16.4	*31.7*	15.8	*31.5*	15.6	*40.7*	11.7	*26.7*	8.1	*32.9*
Spur	3.0	*5.1*	?	*?*	?	*?*	?	*?*	1.8	*4.1*	2.0	*8.1*
Spur/branch height ratio	17%	*–*	?	*–*	?	*–*	?	*–*	15%	*–*	25%	*–*

**Description.** Females (i.e., from the third instar onwards): Body dark red and stout (Fig. 12a), with large ruby eyes not visible after mounting in Hoyer’s medium. Elongated, dactyloid cephalic papillae (secondary clavae) and (primary) clavae. Peribuccal cirri with bulbous cirrophores. Cirrus *A* short, with the proximal end of flagellum smooth and thickened (Fig. 12a).

Dorsal plates thick and strongly sclerotized, with a poorly visible intracuticular sponge layer. Epicuticular granules well separated on all plates except for the lateral portions of the paired segmental plates; granules connected by thick *striae*. Granules are raised, leaving deep pseudopore-like areas between them (Fig. 12a, 15). Cephalic plate large, adjacent to an evident rectangular cervical (neck) plate. Scapular plate without micropores (Fig. 15a). Median plates m1 unipartite and m2 bipartite, with the anterior portion extremely reduced. Median plate m3 rudimentary and smooth centrally. Two pairs of large segmental plates with evident but thin transverse belts. Caudal plate large, with short sclerotized incisions (Fig. 12a).

Ventral cuticle with a pair of subcephalic plates and a pair of trapezoidal genital plates. Uniform ventral wrinkling present. Pedal plates I–III absent, pedal plate IV unsculptured. Leg IV with dentate collar. Pulvini light, clearly visible only under NCM. Spine on leg I (Fig. 12a) and an elongated papilla on leg IV (Fig. 12b) are present. Claws I–III shorter than claws IV. External claws on all legs spurless. Internal claws with short spurs positioned at approximately 1/4 of the claw height and only slightly divergent from main branches.

Males (i.e., from the third instar onwards): Body small and less plump than that of female (Fig. 12). Dorsal sculpturing identical to that in females (Fig. 13a, 14a). Gonopore ovoid, with a semicircular slit and two anteriorly directed and thickened valves (Fig. 13b, 14b).

Juveniles (i.e., the second instar): Gonopore absent; the remaining traits identical to adults.

Larvae (i.e., the first instar): Median plate m3 undeveloped. Dorsal sculpturing disparate from older life stages; belts of endocuticular pillars arranged in an ornamented pattern, especially in the central portions of plates (Fig. 16). Epicuticular granules absent. Gonopore and anus absent.

Eggs: Up to four dark orange eggs in an exuvia.

Type material: Holotype (adult ♀, slide IN.040.01), allotype (adult ♂, slide IN.041.01) and 4 paratypes (1♀, 1♂, 1 juvenile, 1 larva; slides IN.040.02–03, IN.075.01, IN.076.01). Hologenophores: two specimens of unidentified sex (IN.040.04, IN.076.02), one exuvia containing eggs (IN.075.02). Slides IN.040.02–03 deposited in UP, and the remaining slides deposited in UJ.

Type locality: 15°05′44″N, 74°12′41″E, 77 m asl; India, Goa, Western Ghats, Netravali; moss from tree bark, spice plantation.

Additional locality: 14°58′01″N, 74°09′30″E, 100 m asl; India, Goa, Western Ghats, Cotigao; moss from tree bark in forest canopy, moist deciduous forest.

Etymology: From Latin *indicus* = Indian. The name refers to the Indian subcontinent, where the new species was found. An adjective in the nominative singular.

**Fig. 17:**
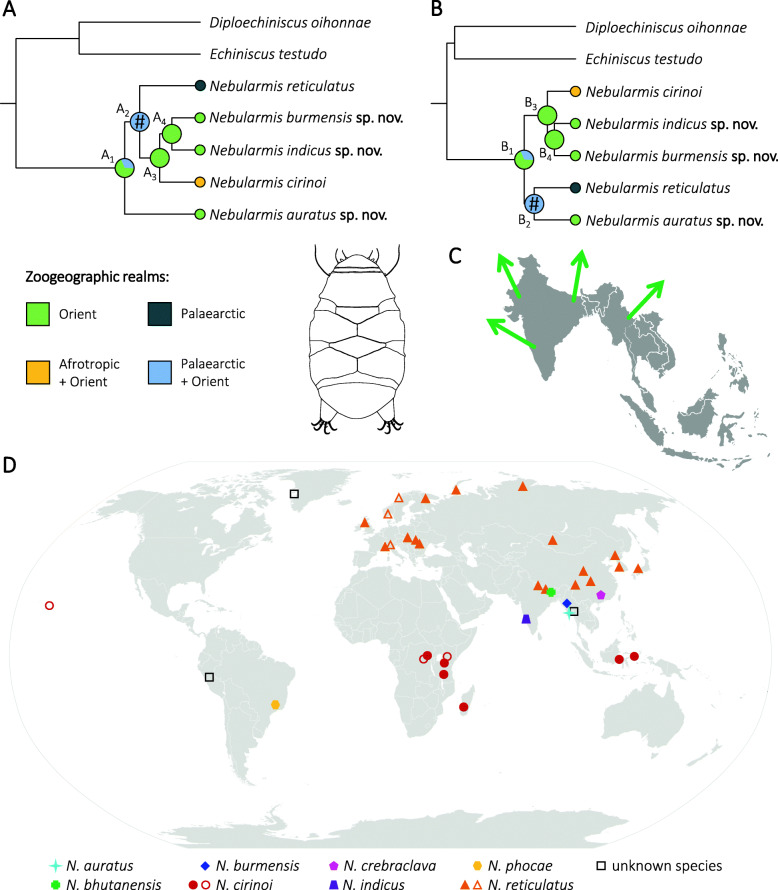
Biogeographic scenarios for the genus *Nebularmis* as inferred in the S-DIVA on the two Bayesian phylogenetic trees: A – under the random local clock with the coalescent tree prior, B – under the random local clock with the speciation: Yule process as the tree prior. A_1_/B_1_–A_4_/B_4_ denote subsequent nodes. *Echiniscus testudo* and *Diploechiniscus oihonnae* were used as outgroups. Hashes (#) signify unsupported nodes, the remaining nodes received maximal (1.00) support; C – schematic hypothetical dispersal from Southeast Asia; D – World map shows records (filled symbols) and likely records (empty symbols) of a given *Nebularmis* species. See [28] for likely records of *N. reticulatus,* and the Discussion herein for analogous reports of *N. cirinoi*

Phylogenetic position. The species was inferred to be a sister species to *Nebularmis burmensis* **sp. nov.** (Fig. 17). The *p*-distances in COI ranged from 9.5% (*N. burmensis* **sp. nov.**, MW178238) to 16.4% (*N. reticulatus*, MN263917). Intraspecific variability ranged from 1.6 to 5.1%. It has been demonstrated that thresholds used in determining barcoding gaps differ greatly between various animal groups [64], often exceeding the arbitrary 3% COI threshold proposed by some studies and commonly applied in DNA-based taxonomy. Our experience suggests that there could be no universal thresholds for all tardigrade groups and that in some of them, the threshold might be higher than that in others [65]. Importantly, systematic testing of intra- and interspecific genetic distances among tardigrade species from different evolutionary lineages is yet to be performed.

See Table 9 for interspecific comparisons among *Nebularmis* (i.e., multiple differential phenotypic descriptions).

**Table 9:** Discriminative criteria used in *Nebularmis* taxonomy (all traits refer to states present in adults if not referred directly to other life stages; comparison based on all examined populations, see also [78, 28] for *N. cirinoi* and *N reticulatus*)

CHARACTERISTIC	*Nebularmis auratus* sp. nov.	*Nebularmis bhutanensis* sp. nov.	*Nebularmis burmensis* sp. nov.	*Nebularmis cirinoi*	*Nebularmis crebraclava*	*Nebularmis indicus* sp. nov.	*Nebularmis reticulatus*
Cephalic papilla	elongated and thin	very large and swollen	elongated and thin	elongated and thin	elongated and thin	elongated and thin	elongated and thin
Primary clava	elongated, reduced	blunt-ended, of average size	elongated, reduced	blunt-ended, of average size	blunt-ended, of average size	elongated, reduced	usually blunt-ended, of average size
Cirrus *A*	17*–*22% of the body length, with smooth and thickened proximal end of flagellum	22*–*26% of the body length, with smooth and slightly thickened proximal end of flagellum	16*–*19% of the body length, with smooth proximal end of flagellum roughly equal in width to the distal end	28*–*35% of the body length, with smooth and slightly thickened proximal end of flagellum	16*–*27% of the body length, with smooth and slightly thickened proximal end of flagellum	19*–*26% of the body length, with smooth and thickened proximal end of flagellum	27*–*63% of the body length, often with rugose proximal end of flagellum roughly equal in width to the distal end
Micropores in the scapular plate	present and randomly distributed (absent in juveniles)	present along anterior and posterior margins	absent	absent	absent	absent	absent or present along anterior and posterior margins (present in juveniles)
Epicuticular granules	tendency towards merging, but still well-separated in some plates	well-separated in all plates, hexagonal and not circular, with evident *striae*	well-separated in median and anterior portions of paired segmental plates, evident bumps in the latter	strong tendency towards merging, circular and very wide	well-separated in all plates, with very wide spaces between them, hexagonal and not circular, with evident *striae*	well-connected by thick *striae* but clearly separated in all plate portions except for the lateralmost portions of paired segmental plates	strong tendency towards merging, usually visible in the scapular plate
Pedal plate IV	unsculptured	strongly sculptured	unsculptured	weakly sculptured	strongly sculptured	unsculptured	unsculptured
Claw branches IV [μm/*sp*]	19.2*–*22.2 *31.6–33.6*	17.3*–*20.1 *34.4–44.9*	18.1*–*20.5 *32.9–37.1*	15.2*–*19.7 *26.6–33.7*	16.1*–*21.4^b^	15.6*–*18.0 *30.7–40.7*	17.5*–*33.9 *29.9–49.4*
Claw spurs	of average size, isonych and with blunt ends	very large and heteronych, spurs IV larger and more divergent from branches than spurs I*–*III	small and short, isonych	of average size and heteronych, spurs IV larger and more divergent from branches than spurs I*–*III	very large, no data available on whether spurs IV differ from spurs I–III	small and short, isonych	large and isonych
Males^a^	unknown	present	unknown	absent	present	present	absent

^a^ Numerous populations of *N. cirinoi* and *N reticulatus* have been examined, and no males were found; thus, the assertion about their parthenogenetic nature is supported. For two other species, *Nebularmis auratus* **sp. nov.** and *Nebularmis burmensis* **sp. nov.**, population sizes were too small to exclude dioecy

^b^ Data not provided in the original description [66]

Proximal end of flagellum = flagellum base

### Phylogeography

Two topologies were observed in the Bayesian analyses based on four datasets (see Materials and methods): ((((*N. auratus* **sp. nov.** (((*N. reticulatus* ((*N. cirinoi* (*N. burmensis* **sp. nov.** + *N. indicus* **sp. nov.**)))) (Fig. 17a) and (((*N. reticulatus* + *N. auratus* **sp. nov.**) + ((*N. cirinoi* (*N. burmensis* **sp. nov.** + *N. indicus* **sp. nov.**))) (Fig. 17b). In both cases, monophyly of the genus was maximally supported, and S-DIVA indicated that Southeast Asia (Indomalayan region) is the ancestral region of *Nebularmis*, only slightly differing in the probability for an Oriental origin (A_1_ = 69%, B_1_ = 65%). Node A_2_ does not conclusively identify the ancestral region for the clade ((*N. reticulatus* ((*N. cirinoi* (*N. burmensis* **sp. nov.** + *N. indicus* **sp. nov.**))), but the clade ((*N. cirinoi* (*N. burmensis* **sp. nov.** + *N. indicus* **sp. nov.**)) was inferred as having an Oriental origin in both trees (A_3_ and B_3_ = 100%), thus implying the dispersal of *N. cirinoi* to the Afrotropics. Node B_2_ is analogously inconclusive regarding the ancestral region for the putative sister species *N. reticulatus* and *N. auratus* **sp. nov.**

## Discussion

### Morphology of *Nebularmis*

**Fig. 18:**
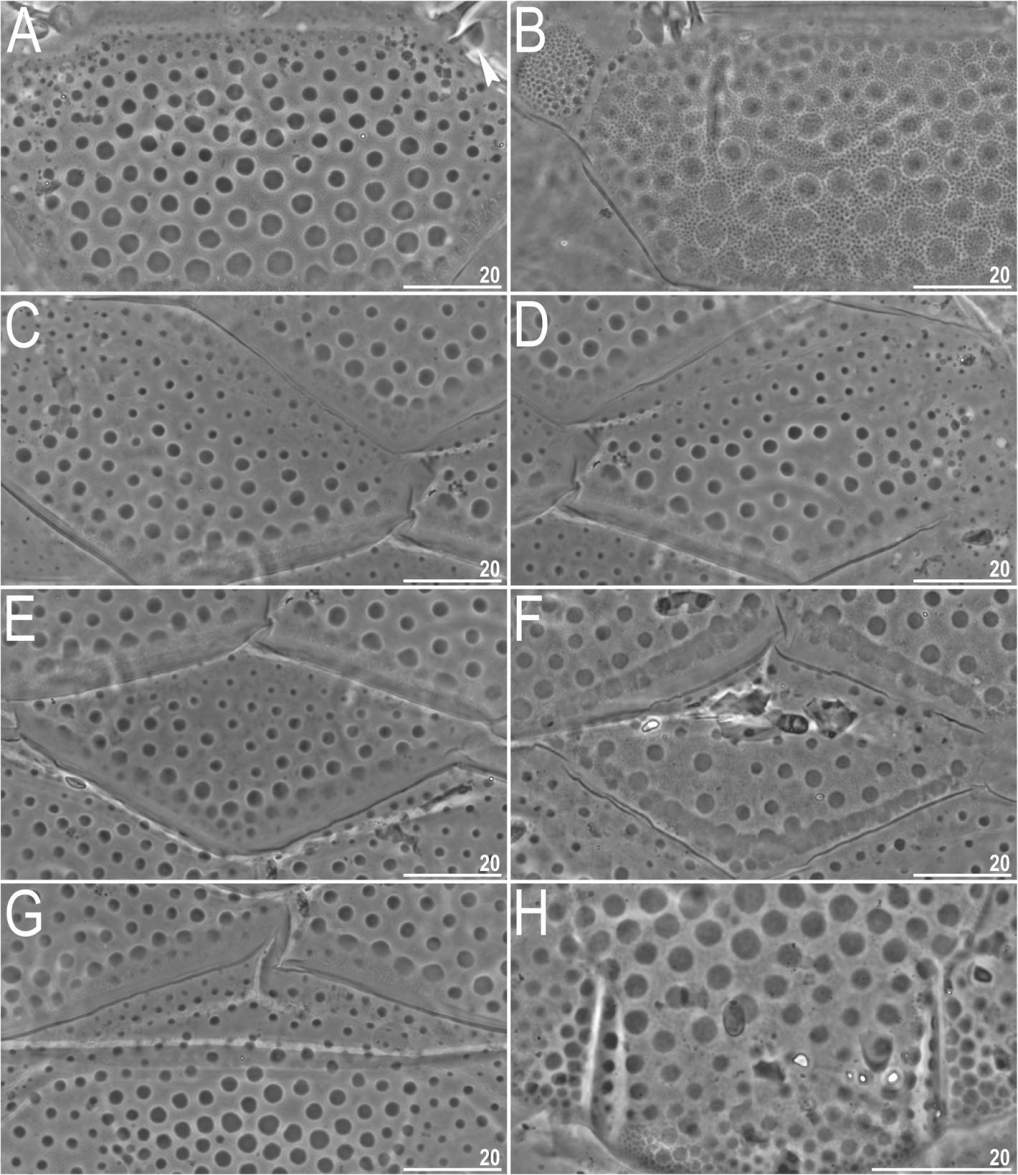
Dorsal plate morphology of *Echiniscus palmai* (PCM): A – surface of the scapular plate (the incised arrowhead indicates the blunt-ended clava), B – inner sponge layer, C–D – paired segmental plates, E–F – second median plate, G – third median plate; H – caudal (terminal) plate with incisions. Scale bars in μm

The original generic diagnosis of *Nebularmis* by Gąsiorek et al. [26] was based on populations of *N. reticulatus* and *N. cirinoi* examined at the time of its erection, supplied by the scarce and mostly dubious data from the original descriptions of other species known at the time. While studying the description of *Claxtonia crebraclava* [66], we noted that the species exhibits an intracuticular sponge layer and that the sculpture resembles that of *N. bhutanensis* **sp. nov.** Thus, we concluded that *Echiniscus crebraclava* was incorrectly transferred to *Claxtonia* by Gąsiorek et al. [26]; herein, we rectify this mistake by designating the species as *Nebularmis crebraclava* **comb. nov.** Although Sun et al. [66] treated the spaces between epicuticular granules as pores, these spaces are not pores; they are simply thinned portions of the cuticle. The original description of the species also states that cephalic papillae are particularly thick, but these structures have a size and shape typical of *Nebularmis* (e.g., compare Fig. 1a in Sun et al. [66] with Fig. 6a, 9a, 10, 12a herein). The discovery of *N. bhutanensis* **sp. nov.** and *N. indicus* **sp. nov.** forces an amendment of the diagnosis of the genus, as new morphological criteria must be introduced to accommodate the expanded variability. Epicuticular granules can be connected by *striae* in *Nebularmis* (*N. crebraclava*, *N. bhutanensis* **sp. nov.** and *N. indicus* **sp. nov.**), as in *Stellariscus* [67] (however, the intracuticular sponge layer separates both genera, as granules are always solid and composed of a uniform cuticular matrix in the latter genus), or granules can be widely spaced, circular and unconnected (*N. burmensis* **sp. nov.**), calling to mind the bumps in *Echiniscus palmai* [68] (Fig. 18a, c–h). In fact, *E. palmai* shares some characteristics of *Nebularmis*: the intracuticular matrix is similar to the sponge layer (Fig. 18b), subcephalic plates are present (genital plates were not mentioned in the original description and are not discernible in the examined specimens due to retracted legs IV), and claws are long, firm and sabre-like. Moreover, dorsal bumps also tend to merge in the posterior portions of some plates (Fig. 18f). Based on these premises, we hypothesize that this species is related to *Nebularmis* or perhaps should be incorporated into this genus. The argument against its inclusion in *Nebularmis* involves the presence of weakly sclerotized stylet supports, but presently it is difficult to determine whether this taxonomic criterion is homogenous in every monophyletic echiniscid genus, especially within the *Echiniscus* line [34].

**Fig. 19:**
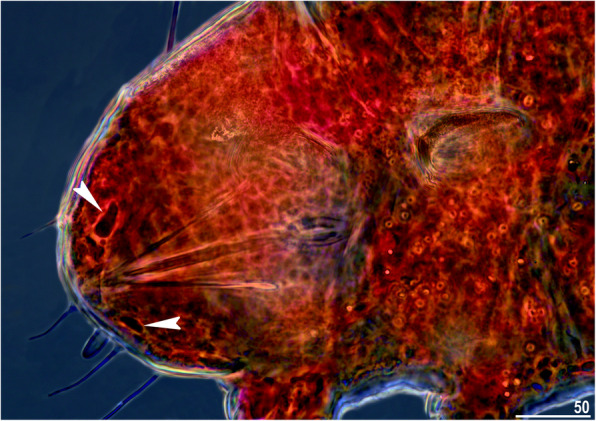
A living specimen of *Nebularmis reticulatus* (population IT.126 from [28]) showing large red eyes. Scale bar in μm

*Stellariscus*, with characteristic black pigmented eyes [67], is generally easily distinguishable from red-eyed *Nebularmis* when specimens are observed *in vivo* (Fig. 19). However, eye pigmentation can dissolve rapidly in the smallest individuals after mounting in Hoyer’s medium. In addition to the abovementioned difference in dorsal sculpturing, the ventral cuticle is also divergent in both genera. In *Nebularmis*, wrinkling is regular and continuous, whereas the ventral plates of *Stellariscus* are autapomorphic, with two distinct types of which one type has nondistinctive outer margins when observed under LCM. Nevertheless, the morphological and reproductive similarities between *Nebularmis* and *Stellariscus* should be considered superficial, as the latter genus probably occupies a more basal position within the *Echiniscus* lineage [4]. The finding of males of two new *Nebularmis* species was unexpected, although not unheard of in the history of echiniscid taxonomy; for example, *Echiniscus* was once considered exclusively parthenogenetic [69]. In fact, despite being one of the first described tardigrade genera [31], *Echiniscus* males were detected only at the end of the XX century [69–72].

### Taxonomic key

**Fig. 20:**
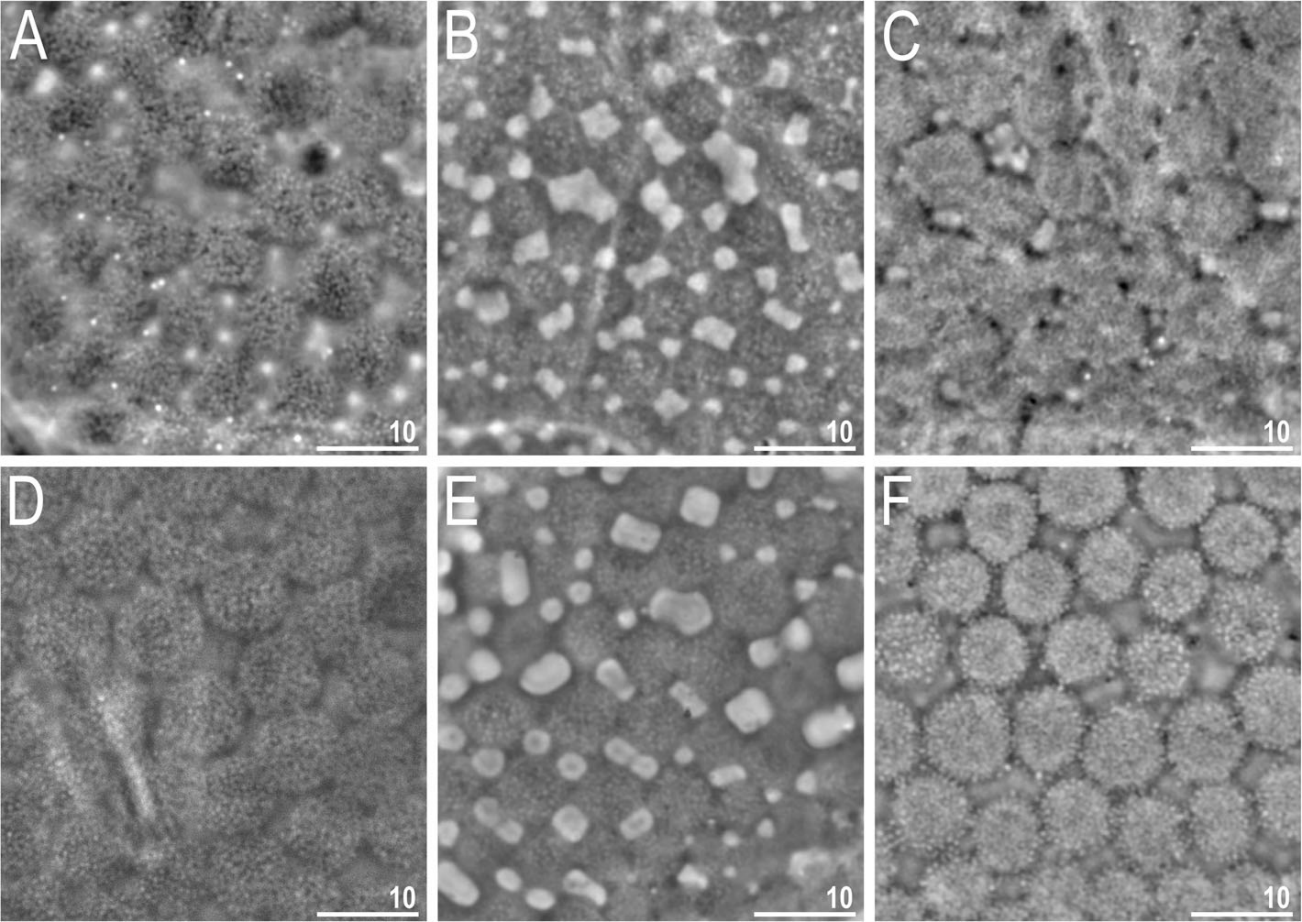
Interspecific variability in scapular plate sculpturing in *Nebularmis* (PCM): A – *N. auratus ***sp. nov.**, B – *N. bhutanensis ***sp. nov.**, C – *N. burmensis ***sp. nov.**, D – *N. cirinoi*, E – *N. indicus ***sp. nov.**, F – *N. reticulatus*. Scale bars in μm

The key provided below is mostly based on qualitative traits of sexually mature (adult) individuals. This is a usual practice in modern echiniscid taxonomy, as previous authors did not distinguish between different life stages. Consequently, larvae and juveniles are excluded from the key since they are not known for every species. Furthermore, representatives of *Nebularmis* designated in [26, 28] as *nomina dubia* (i.e., *N. carsicus*, *N. mihelcici*, *N. nobilis*, *N. tardus*) or *nomina inquirenda* (*N. japonicus*, *N. markezi*, *N. phocae*) or junior synonyms (*N. mihelcici*) were also excluded. Importantly, the generic affinity of *N. markezi* is uncertain since eye pigment in this species was described by Mihelčič [73] as black-red to black, whereas all *Nebularmis* specimens we examined before mounting in Hoyer’s medium had red eyes, and no variability in this trait has been detected within any of the known echiniscid genera [34] (i.e., if present, eyes are either red or black). Thus, although the sculpture is similar to that of *Nebularmis* (see Fig. 20 for direct comparisons between scapular plates of *Nebularmis* spp.), we excluded this species from the key until this ambiguity is resolved.

1. *Striae* between epicuticular granules present ..................2

–. *Striae* between epicuticular granules absent ...................4

2(1). Pedal plates IV sculptured .................................................3

–. Pedal plates IV unsculptured ................................................................................................ *Nebularmis indicus* **sp. nov.**

3(2). Secondary clavae greatly enlarged, micropores present in the scapular plate .................................................................................................................. *Nebularmis bhutanensis* **sp. nov.**

–. Secondary clavae not enlarged, micropores absent in the scapular plate ..................................................................................................................... *Nebularmis crebraclava* (Sun et al., 2014)

4(1). Claw spurs heteronych ............................................................................................ *Nebularmis cirinoi* (Binda & Pilato, 1993)

–. Claw spurs isonych ...........................................................5

5(4). Claw spurs with blunt ends.................................. .................................................... *Nebularmis auratus* **sp. nov.**

–. Claw spurs with acute ends ...................................................6

6(5). Granules in the anterior portions of paired segmental plates in the shape of prominently convex bumps that are clearly separated; cirri *A* < 20% of the body length .................................. *Nebularmis burmensis* **sp. nov.**

–. Granules in anterior portions of paired segmental plates flattened and often merged; cirri *A* > 25% of the body length .......................................................................................................................*Nebularmis reticulatus* (Murray, 1905)

### Biogeography of *Nebularmis* in the context of echiniscid phylogeny

The oldest record of *Nebularmis* in Asia is that of Murray [74]. He reported *Nebularmis reticulatus* from the Sikkim Himalayas, and the presence of this typical Palearctic species in this region cannot be dismissed since it was reported from the neighboring Nepal [75] and later reaffirmed during the formal redescription and re-examination of those specimens [28]. Since the Indian subcontinent and Indochina are characterized by subtropical and tropical climates, in contrast to the typical mountainous or high boreal records of this species, it seems justified to hypothesize that the Himalayas most likely define the southernmost boundary of the *N. reticulatus* range. Specimens representing *Nebularmis* were found in Greenland [36], but the present condition of those slides does not allow confirmation of their affinity to *N. reticulatus*. Even if *N. reticulatus* was indeed recorded in Greenland, anthropogenic dispersal would seem to be the most likely explanation. Although Greenland is traditionally regarded as a part of the Nearctic, its invertebrate fauna is largely a mixture of Palearctic, cosmopolitan and endemic taxa [76, 77]; thus, the eventual presence of *N. reticulatus* in Greenland would not affect the status of a principally Palearctic species [28]. *Nebularmis* is certainly present in the Neotropical region [29, 36], but the taxonomic identity of South American individuals remains obscure. *Nebularmis phocae* requires a redescription [28], and there is a chance of conspecificity with *N. cirinoi* [63] since we documented a wide distribution of this species in the African and Asian tropics and the species may be pantropical. The specimen from the Dastych collection originating from the Andes and representing *Nebularmis*, which we examined, is different from all other *Nebularmis* spp. by its elongated and thin claws; thus, it most likely represents a new species.

*Nebularmis* specimens from the Ruwenzori Mountains (Central Africa) and Doi Inthanon (Chiang Mai, Northern Thailand) are unidentifiable due to their poor states. The dorsal plate sculpturing of the African material clearly resembles *N. cirinoi* and these specimens likely belong to this taxon, as each location from which specimens were reported was in a mountainous tropical rainforest (this study, [78]). An undetermined species, most closely resembling *N. cirinoi* (misidentified as a *Viridiscus* in Tsaliki et al. [79]), inhabits the Hawaiian Archipelago. Consequently, *Nebularmis*, with records throughout the world, has a very wide geographic distribution, and although it has not been reported from the Nearctic and Australasia [32], we hypothesize that these gaps in its cosmopolitan distribution are most likely due to undersampling in these regions combined with the rarity of the genus.

Southeast Asia has an intricate geological history, with the Indian subcontinent and Indochina considered a part of the Gondwanan paleocontinent that later joined the Laurasian Palearctic region [80–82]. *Nebularmis* is inferred herein as having an Oriental ancestry, which potentially renders this region vital for echiniscid diversification. However, Southeast Asia belongs to one of the worst-sampled areas of the globe, which is evidenced, for example, by the fact that three out of the four new *Nebularmis* species described in this study are also the first tardigrade records for Bhutan and Myanmar. As many post-Gondwana taxa seem basal with respect to the derived Laurasian fauna, they are important in the understanding of heterotardigrade phylogeny [34, 83, 84]. In fact, the number of Laurasian echiniscid genera is much higher than that of Gondwanan genera, at 6:3 (*Diploechiniscus*, *Proechiniscus*, *Multipseudechiniscus*, *Novechiniscus*, *Parechiniscus* and *Cornechiniscus*, the last likely dispersed to Africa and South America, vs *Antechiniscus*, *Mopsechiniscus* and *Barbaria*, the last of which contains one species that dispersed to the southern Nearctic region [85]). As Southeast Asia is not as geologically old as South America and Australia, the presented hypothesis relating to the Oriental origin of *Nebularmis* involves its relatively recent divergence and radiation away from the current centers of relic Gondwanan tardigrade faunal diversity [34, 86].

### Spurious taxa

Tardigrade taxonomy is burdened with the same problems as encountered in other meiofaunal groups that underwent preliminary study in the XVIII and XIX centuries, i.e.: a lack of type series, species descriptions considered by modern standards as insufficient and/or inadequate, difficulties with discerning intraspecific from interspecific variability, and lack of type DNA sequences [4, 7–10, 28, 87]. The state-of-the-art in phyla characterized by a much younger taxonomic history, such as Loricifera [88], is enviable compared to that in tardigrades. The first described representatives of these groups were analyzed in much greater detail, which allowed for a high resolution of detection of species diversity and reliable subsequent records of early-described species [89]. At the 13^th^ Symposium on Tardigrada in Modena, mirroring the actions of rotiferologists [35], a proposal to form a list of available names for this phylum in accordance with the rules of ICZN was put forward. There is a compelling need for the creation of such a list, as even a quick glance at the current checklist of tardigrade taxa, with a number of *nomina inquirenda* and *species dubia* embedded therein [22], reveals that significant problems exist with the reliability of species descriptions in numerous genera. *Nebularmis* can serve as a perfect example; of the eight valid species listed in the last edition of the checklist, no taxonomic obscurities were identified in only two species. The remaining six species are indistinguishable [26, 28]; thus, the most practical thing to do would be to abandon using these names once and for all. Therefore, we urge the international community of tardigradologists to intensify actions leading to the uncluttering and sorting of tardigrade taxonomy. This will prevent nontaxonomists unacquainted with the meanders of systematics from using dubious names, e.g., in local checklists or ecological and experimental studies.

## Conclusions

*Nebularmis* represents yet another echiniscid genus in which dorsal sculpturing and claw morphology are crucial taxonomic criteria [4, 26, 28, 34, 90–93]. Despite some similarities between *Nebularmis* with *Stellariscus*, the two genera are not directly related. Continental Asia is an important but mostly undersampled region in the context of echiniscid diversity and phylogeny. Finally, we propose enhancing efforts to eliminate dubious and/or unidentifiable water bear species from the modern professional literature by creating a list of available names.

## Supplementary Information

**Additional file 1. **Primers and DNA amplification protocols.

**Additional file 2. **18S rRNA alignment.

**Additional file 3. **28S rRNA alignment.

**Additional file 4. **ITS-1 alignment.

**Additional file 5. **ITS-2 alignment.

**Additional file 6. **COI alignment.

**Additional file 7. **Concatenated 18S + 28S + ITS alignment used for the biogeography reconstructions.

## Data Availability

All data generated or analyzed during this study are included in the article, with supplementary information files providing detailed phylogenetic datasets. Genetic data are deposited in GenBank.
